# Efficacy and safety of once‐weekly basal insulin therapy in people with type 1 diabetes: A systematic review and meta‐analysis

**DOI:** 10.1111/dom.70176

**Published:** 2025-10-06

**Authors:** Ludovico Di Gioia, Sergio Di Molfetta, Irene Caruso, Mariangela Caporusso, Gian Pio Sorice, Angelo Cignarelli, Annalisa Natalicchio, Sebastio Perrini, Luigi Laviola, Francesco Giorgino

**Affiliations:** ^1^ Department of Precision and Regenerative Medicine and Ionian Area, Section of Internal Medicine, Endocrinology, Andrology and Metabolic Diseases University of Bari Aldo Moro Bari Italy; ^2^ Endocrinology Unit Regional General Hospital “Francesco Miulli” Bari Italy; ^3^ Endocrinology Unit University Hospital “Consorziale Policlinico” of Bari Bari Italy; ^4^ Section of Endocrinology, Department of Medicine and Surgery LUM University Casamassima (BA) Italy

**Keywords:** basal insulins, hypoglycaemia, insulin therapy/therapies, meta‐analysis, type 1 diabetes

## Abstract

**Aims:**

Once‐weekly basal insulins may offer similar or superior HbA1c reduction compared to once‐daily analogues in people with type 1 or type 2 diabetes. However, concerns about hypoglycaemia persist in individuals on multiple daily injections. This meta‐analysis (PROSPERO CRD42024606874) aimed to evaluate the efficacy and safety of once‐weekly basal insulin therapy in type 1 diabetes.

**Materials and Methods:**

A systematic search was conducted in MEDLINE, Web of Science and CENTRAL up to 1 April 2025. We included randomized controlled trials (RCTs) comparing insulin icodec or efsitora against once‐daily basal insulins in people with type 1 diabetes. Three reviewers independently evaluated the retrieved citations. The primary outcome was the change in HbA1c. Meta‐analysis was performed using fixed‐ or random‐effects models based on heterogeneity.

**Results:**

Five RCTs were included, enrolling 1629 adults living with type 1 diabetes. Once‐weekly and once‐daily basal insulins had similar effects on HbA1c (high certainty), body weight (moderate certainty), time in range (moderate certainty) and time above range. However, safety concerns emerged due to increased rates of level 3 hypoglycaemia (incidence rate ratio 2.532, 95% confidence interval [CI] 1.758–3.645; moderate certainty). A significantly lower weekly bolus insulin dose was observed with once‐weekly basal insulin therapy (estimated treatment ratio 0.837, 95% CI 0.794–0.882, *I* ^2^ = 0%; high certainty).

**Conclusions:**

This meta‐analysis is the first to evaluate the efficacy and safety of once‐weekly basal insulin therapy exclusively in adults with type 1 diabetes and including all published RCTs. The analysis demonstrated a similar glucose‐lowering effect compared to once‐daily basal insulin but revealed an increased occurrence of severe hypoglycaemia.

## 1 INTRODUCTION

Since its introduction in the 1920s, insulin therapy has been the cornerstone of treatment for type 1 diabetes. Above all, the advent of once‐daily basal analogues has resulted in similar or improved glycaemic control with reduced risk of hypoglycaemia, the latter being even more pronounced with second‐generation analogues, compared to earlier formulations.^1^, ^2^, ^3^, ^4^, ^5^, ^6^ Despite these innovations, achieving and maintaining optimal glucose levels remains a significant challenge for many individuals with type 1 diabetes, with only a relatively small percentage meeting the desired glycaemic targets.^7^ Moreover, inconsistent administration of daily basal insulin has been reported in up to 22% of people with type 1 diabetes, with unfavourable effects on glycaemic control, glycaemic variability and occurrence of diabetic ketoacidosis (DKA).^8^, ^9^

Very recently, a third generation of basal insulin analogues has been developed with substantial changes to human insulin structure and prolonged duration of action, allowing for once‐weekly administration.^10^ While different weekly insulin formulations have been proposed, insulin icodec and insulin efsitora alfa (efsitora, also known as basal insulin Fc, BIF) are the furthest along in the evaluation process, and the former has been recently approved for clinical use in some countries.^11^ The introduction of once‐weekly basal insulins represents a promising opportunity to achieve glycaemic control while dramatically reducing the number of basal insulin injections required annually. Indeed, phase 2 and phase 3 clinical trials have demonstrated that once‐weekly basal insulins are similarly or more effective than once‐daily basal insulins in providing glycated haemoglobin (HbA1c) reduction in people with type 1 and type 2 diabetes.^12^, ^13^, ^14^ However, concerns about the risk of hypoglycaemia persist in individuals on a multiple daily insulin injections regimen.^14^, ^15^, ^16^ This systematic review and meta‐analysis aims to summarize efficacy and safety data of once‐weekly as compared with once‐daily basal insulins in individuals with type 1 diabetes.

## 2 METHODS

The study protocol was registered prior to conduct (PROSPERO CRD42024606874).

### 2.1 Data sources

We searched MEDLINE (via Ovid), Web of Science and CENTRAL from inception to April 1, 2025 (Appendix S1) and performed hand‐searching in PubMed to identify online publications ahead of print.

### 2.2 Study selection

We included randomized clinical trials (RCTs) enrolling people with type 1 diabetes treated with insulin injections, evaluating a once‐weekly basal insulin against any once‐daily basal insulin and reporting any outcome of interest. Trials conducted in special populations of patients (pregnant women, patients with kidney or liver failure, hospitalized patients or highly unstable diabetes), or evaluating patients with any other type of diabetes, were excluded as insulin therapy in these subgroups of patients and/or situations may be influenced by several factors not reflecting usual practice.

The primary review outcome was the change in HbA1c from baseline (mean difference [MD], 95% confidence interval [CI] or estimated treatment difference [ETD], 95% CI). Secondary outcomes included changes in fasting plasma glucose (FPG), body weight and body mass index, mean daily basal insulin dose, total daily insulin dose, time in range (TIR, 70–180 mg/dL), time below range (TBR, <70 mg/dL), time above range (TAR, >180 mg/dL), measures of glucose variability (coefficient of variation of mean glucose [CV]), incidence of severe hypoglycaemic events, incidence of DKA, patients' satisfaction and quality of life. All causes of death, occurrence of major adverse cardiovascular events, lower limb gangrene or lower limb amputation, development/worsening of diabetic retinopathy or nephropathy and need for kidney replacement therapy were also collected.

### 2.3 Data extraction

Three reviewers (IC, LDG, SDM) independently evaluated the retrieved citations based on predetermined inclusion and exclusion criteria. Disagreements were settled by discussion or by a third party (MC, GPS). The following data were collected from the included papers: study characteristics (study design, duration, year of publication, sample size), participants' characteristics (age, sex, HbA1c at baseline, eGFR [mL/min] and disease duration), once‐weekly basal insulin under evaluation, comparator(s), change in HbA1c (%), change in FPG (mg/dL), changes in body weight (kg), mean weekly basal insulin dose (UI), mean weekly total insulin dose (UI), mean daily bolus insulin dose (UI), mean TIR (%), TBR (%), TBR (%) level 1 (54–69 mg/dL) and level 2 (<54 mg/dL), TAR (%), TAR level 1 (181–250 mg/dL) and level 2 (>250 mg/dL), CV (%), patient‐reported outcome measures (PROMs), prevalence/incidence of severe (level 3) hypoglycaemic events and DKA and other severe adverse events (all causes of death, occurrence of major adverse cardiovascular events, lower limb gangrene or lower limb amputation, development/worsening of diabetic retinopathy or nephropathy and need for renal replacement therapy). If the 90% CI was reported, the 95% CI was calculated by adjusting the margin of error based on the standard error (SE) as follows: 95% CI equals ETD plus or minus 1.96 times SE, where SE was derived from the 90% CI. If SE was not reported, it was calculated based on the reported CI limits. For a 95% CI, SE equals the difference between the CI upper and CI lower divided by 2 times 1.96. If a 90% CI was reported, SE was calculated using the difference between the CI upper and CI lower divided by 2 times 1.645. If none of this information was available, the standard deviation (SD) was imputed using the highest SD among the studies included in the meta‐analysis. The SE was then derived using the formula: SE equals SD divided by the square root of the sum of the reciprocals of the sample sizes in the experimental and control groups.^17^ If not provided, the hypoglycaemia rate was calculated as the number of hypoglycaemic events divided by the total patient‐years of exposure in each treatment group, where patient‐years were derived by multiplying the number of patients by the duration of the study in years. If the estimated treatment ratio (ETR) for insulin doses between groups was not directly reported, it was derived from the ETD and the mean weekly dose of the reference group. The ETR was calculated using the formula: ETR = 1 + (ETD/mean weekly dose of the reference group). The 95% CI for the ETR was derived by applying the same formula to the lower and upper limits of the CI for the ETD. Finally, the SE of the ETR was calculated from the 95% CI using the formula: SE = (upper limit of ETR − lower limit of ETR)/(2 * 1.96). Disagreements in data extraction were settled by debate or with the aid of a third party (FG).

### 2.4 Risk of bias assessment

Risk of bias was assessed independently by two reviewers (IC, LDG) through the Cochrane Collaboration's tool (RoB 2, version 22 August 2019; RoB 2 crossover, version 18 March 2021) evaluating the following domains: randomization process; bias arising from period and carryover effects; deviations from intended intervention; missing outcome data; measurement of the outcome; selection of the reported result; overall bias. Each domain was deemed at low risk with some concerns or high risk of bias. Any differences in assessment were resolved by consensus. Certainty of evidence was assessed using the Grading of Recommendations, Assessment, Development and Evaluations (GRADE)^18^ approach using GRADEpro Guideline Development Tool (GRADEpro GDT).^19^

### 2.5 Statistical analysis

The systematic review and meta‐analysis were performed in line with recommendations from the Cochrane Collaboration and the Preferred Reporting Items for Systematic Reviews and Meta‐Analysis (PRISMA) statement guidelines. Data for continuous variables are expressed as mean (SD) or, if variables were not normally distributed, as median (interquartile range); categorical variables were represented as counts or frequencies. Continuous outcomes were assessed using ETD or ETR, whilst binary outcomes were assessed using incidence rate ratio (IRR) or estimated rate ratio (ERR) with their 95% CI. In addition, 95% prediction intervals (PIs) were included in the forest plots to illustrate the range within which the relative treatment effect of a future study is expected to fall, accounting for between‐study heterogeneity. A continuity correction of 0.1 was added to all arms in studies that reported zero events. In addition, heterogeneity was evaluated with Cochran's *Q* test and *I* ^2^: *p*‐values <0.05 and *I* ^2^ > 25% were considered significant for heterogeneity. We used a fixed‐effect model for endpoints with *I* ^2^ < 25% (low heterogeneity) and a random effects model with Hartung–Knapp correction for endpoints with *I* ^2^ > 25% (high heterogeneity) in order to make the results more reliable.^20^ Additionally, both models were applied as a sensitivity analysis to assess the robustness of the findings. Funnel plot and Egger's test when feasible were employed to evaluate the presence of publication bias. A prespecified subgroup analysis was conducted based on the different types of daily or weekly basal insulin analogues used as comparators. The Instrument for assessing the Credibility of Effect Modification Analyses tool was used to assess credibility of subgroup effects for interaction *p*‐values <0.1. All analyses were performed using Rstudio and R package meta.^21^

## 3 RESULTS

### 3.1 Study characteristics

**FIGURE 1:**
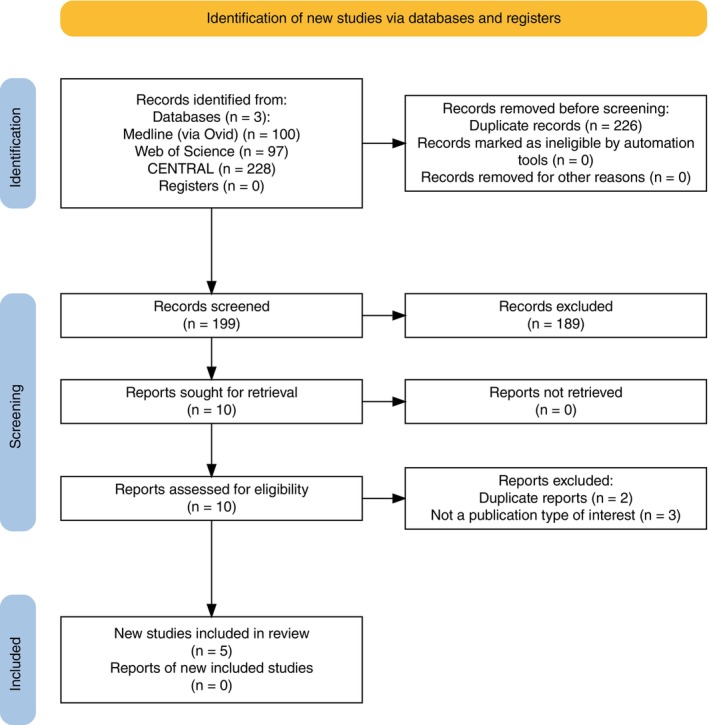
Preferred Reporting Items for Systematic Reviews and Meta‐Analysis (PRISMA) flow diagram of study search strategy. *Source*: Haddaway et al.^22^

The literature search yielded 425 records. After removal of duplicates, 199 abstracts and nine full‐text articles were reviewed, of which 5 were ultimately found to be eligible and included in the meta‐analysis. The selection process is reported in detail in the PRISMA flow diagram (Figure 1). All five studies were conducted in adult populations. Three out of five studies, two phase 1 RCTs versus insulin glargine U100^23^, ^24^ and one phase 3 RCT versus insulin degludec (ONWARDS 6^16^), evaluated the efficacy and safety of insulin icodec, while the remaining two studies, one phase 2^25^ and one phase 3 RCT (QWINT‐5^14^), both versus insulin degludec, evaluated insulin efsitora. Overall, the data of 1629 people were analysed: 862 were included in the once‐weekly basal insulin group and 851 in the once‐daily basal insulin group. Study and patient characteristics are presented in Appendix S2. The overall risk of bias for the main outcome (HbA1c) was deemed low for two trials and of “some concern” for one trial due to deviations from the intended interventions (Appendix S3). Funnel plots did not suggest the presence of publication bias for HbA1c, BW, hypoglycaemia level 3, TIR, TAR, total insulin dose and bolus insulin dose (Appendix S4). Certainty of evidence for the main outcomes is presented in the summary of findings table (Appendix S6).

### 3.2 Effect on HbA1c and body weight

**FIGURE 2:**
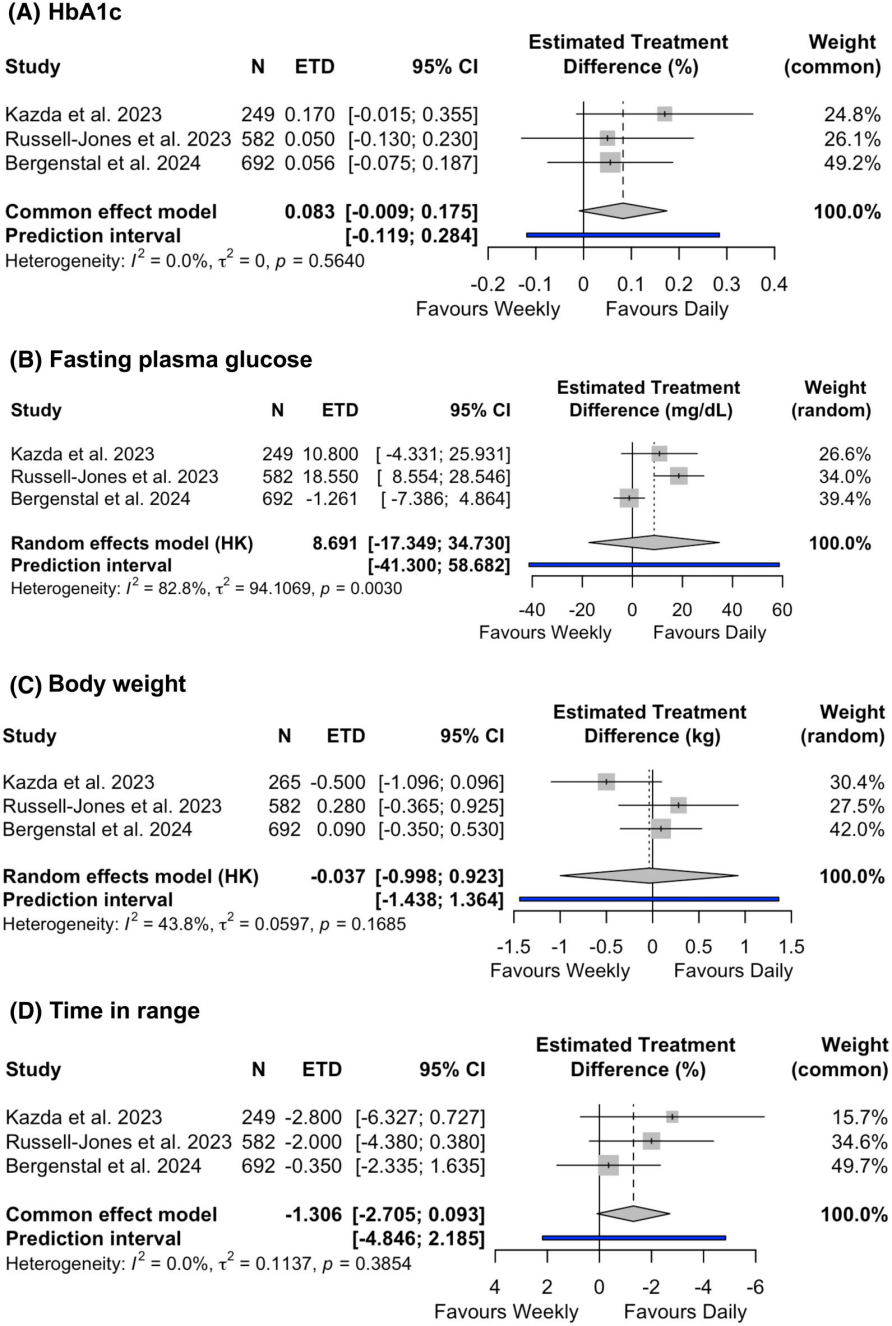
Forest plots summarizing the effects of once‐weekly compared with once‐daily basal insulins on glycated haemoglobin (A), fasting plasma glucose (B), body weight (C) and time in range (D).

Three out of five studies, all versus insulin degludec and including a total of 1523 people, investigated the effect of once‐weekly and once‐daily basal insulin therapy on overall glycaemic control. The ETD on HbA1c between the groups was 0.083% (95% CI −0.009 to 0.175, *I* ^2^ = 0%; high certainty), indicating similar HbA1c lowering efficacy in the two groups (Figure 2A). The subgroup analyses evaluating HbA1c reduction with insulin icodec versus insulin degludec (ETD 0.05%, 95% CI −0.13 to 0.23) and with insulin efsitora versus insulin degludec (ETD 0.094%, 95% CI −0.013 to 0.201) confirmed the results of the overall analysis (Appendix S5.1). The ETD on FPG was 8.7 mg/dL (95% CI −17.3 to 34.7, *I* ^2^ = 82.8%; very low certainty), without significant differences between the groups, albeit with substantial heterogeneity across studies (Figure 2B). However, in the subgroup analysis by type of weekly insulin, insulin icodec achieved lower FPG reduction compared to once‐daily insulins (ETD 18.6 mg/dL, 95% CI 8.7–28.7), while insulin efsitora showed a similar FPG lowering effect (*p* < 0.05 for subgroup differences, Appendix S5.2).

The change in body weight was reported in three out of five studies, including a total of 1539 people, and was not statistically different between once‐weekly and once‐daily basal insulin therapy (ETD −0.037 kg, 95% CI −0.998 to 0.923, *I* ^2^ = 43.8%; moderate certainty), albeit with high heterogeneity across studies (Figure 2C). Similarly, no significant difference was observed in the subgroup analysis evaluating insulin icodec versus insulin degludec (ETD 0.28 kg, 95% CI −0.365 to 0.925) and insulin efsitora versus insulin degludec (ETD −0.169 kg, 95% CI −3.89 to 3.552) regarding body weight changes (Appendix S5.3).

### 3.3 Continuous glucose monitoring

CGM‐derived glucose metrics were available in the three trials versus insulin degludec, including 1523 people. Once‐weekly and once‐daily basal insulins achieved similar improvements in TIR (ETD −1.306%, 95% CI −2.705 to 0.093, *I* ^2^ = 0% moderate certainty; Figure 2D) and TAR (ETD 0.978%, 95% CI −2.686 to 4.641, *I* ^2^ = 19%). Subgroup analyses were in line with the overall results (Appendices S5.4 and S5.5).

Of note, no study reported total TBR, and three out of five studies did not report the CV; therefore, a meta‐analysis could not be performed for these outcomes. Moreover, information on outcome variability was not available for TAR level 1, TAR level 2, TBR level 1 and TBR level 2 in two studies; accordingly, a meta‐analysis was not performed^17^ and available data were summarized in Appendices S5.6–5.9.

### 3.4 Hypoglycaemic events

**FIGURE 3:**
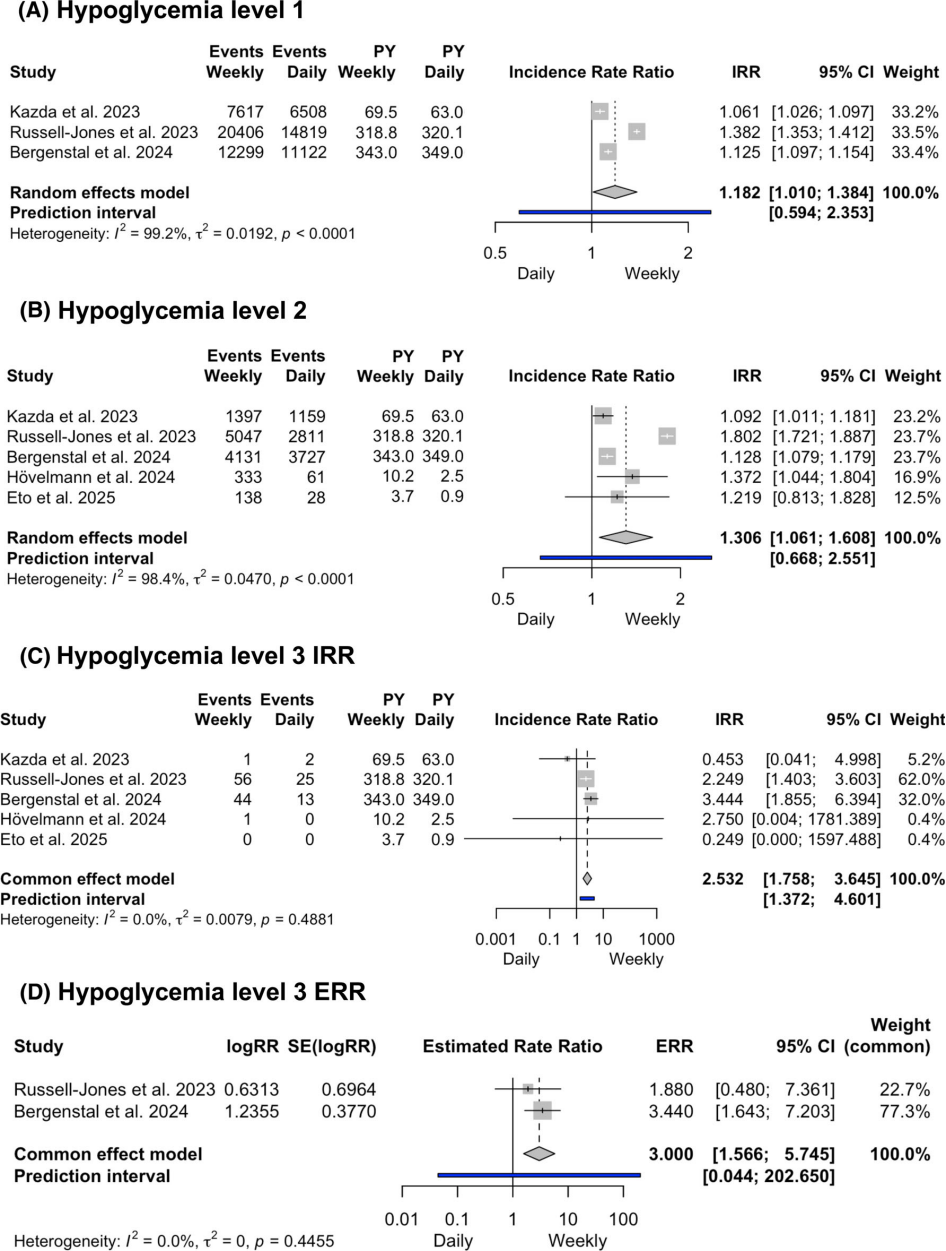
Forest plots comparing the incidence rate of level 1 (A), level 2 (B) and level 3 (C) hypoglycaemic events, as well as the risk ratio for level 3 hypoglycaemic events (D) for once‐weekly compared to once‐daily basal insulins.

The incidence rate (events per patient‐year) of level 2 and level 3 hypoglycaemic events was reported in all five studies (1629 people), while level 1 events were only reported in the three studies versus insulin degludec (1539 people). The rate of level 1 events was significantly higher with once‐weekly compared to once‐daily insulins (IRR 1.182, 95% CI 1.01–1.384, *I* ^2^ = 99.2%; Figure 3A), and the results of subgroup analyses were consistent with the pooled findings (Appendix S5.10). The rate of level 2 events appeared also higher with once‐weekly than with once‐daily insulins (IRR 2.532, 95% CI 1.758–3.645; very low certainty; Figure 3B). However, while subgroup analyses by type of weekly insulin were consistent with the overall analysis, those by type of daily insulin revealed that the incidence rate of hypoglycaemia with weekly insulins was similar to degludec and higher compared to glargine U100 (Appendix S5.11). In line with what was observed for level 1 and level 2 events, the rate of level 3 events was also significantly higher with once‐weekly compared to once‐daily basal insulin therapy (IRR 2.532, 95% CI 1.758–3.645, *I* ^2^ = 0%, moderate certainty, 95% PI 1.372–4.601; Figure 3C and Appendix S5.12). No significant differences were found at subgroup analyses by type of weekly insulin and comparators (Appendix S5.12). Of note, for the phase 3 studies (1274 people in total), ERRs for level 3 hypoglycaemia between treatments were explicitly provided, showing a significantly higher risk of events with the once‐weekly basal insulin therapy at the common effect model meta‐analysis (ERR 3, 95% CI 1.566–5.745, *I* ^2^ = 0%; Figure 3D). A random‐effects model was additionally performed as a sensitivity analysis, yielding consistent point estimates but with a wider CI, reflecting the limited number of events (ERR 3, 95% CI 0.121–74.642, *I* ^2^ = 0%; Appendix S5.12).

### 3.5 Other severe adverse events

In line with our study protocol, occurrence of DKA, death, major adverse cardiovascular events, lower limb gangrene or amputation, development/worsening of diabetic retinopathy or nephropathy and need for kidney replacement therapy were also collected (Appendix S7). However, due to their very low incidence, heterogeneous reporting across studies and occasional lack of treatment‐relatedness, these events were not included in quantitative meta‐analysis.

### 3.6 Insulin dose and treatment satisfaction

**FIGURE 4:**
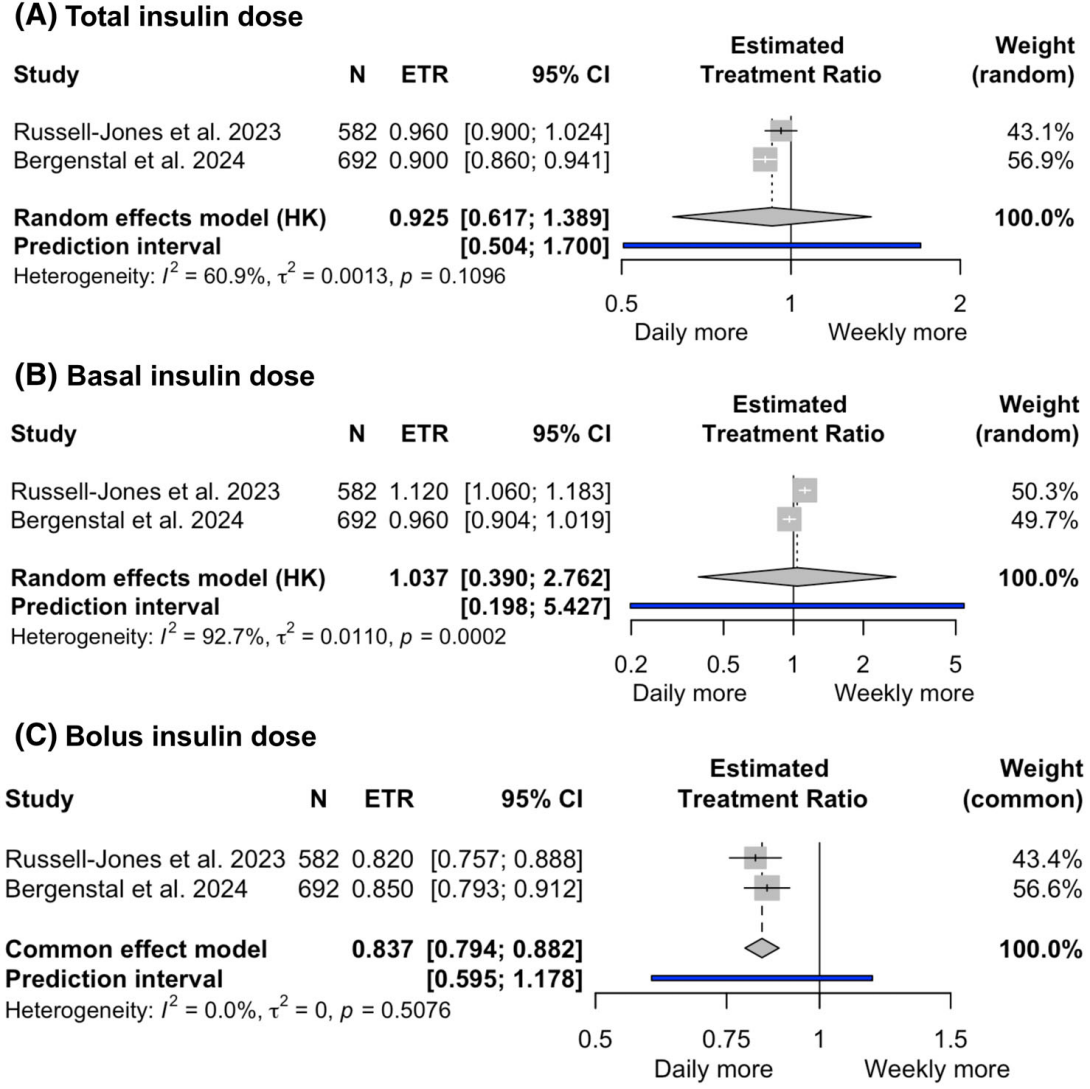
Forest plots summarizing total insulin dose (A), basal insulin dose (B) and bolus insulin dose (C) with once‐weekly compared with once‐daily basal insulins.

The two phase 3 studies (1274 people) also reported weekly total insulin dose, basal insulin dose, bolus insulin dose and treatment satisfaction. There was no difference in weekly total (ETR 0.925, 95% CI 0.617–1.389; *I* ^2^ = 60.9%) (Figure 4A) and basal (ETR 1.037, 95% CI 0.39–2.762; *I* ^2^ = 92.7%) (Figure 4B) insulin doses between once‐weekly and once‐daily basal insulin groups. Both comparisons showed high heterogeneity across studies, which should be taken into account when interpreting these findings. In contrast, a significantly lower weekly bolus insulin dose was observed with once‐weekly basal insulin therapy (ETR 0.837, 95% CI 0.794–0.882, *I* ^2^ = 0%; high certainty) (Figure 4C and Appendix S5.15).

Treatment satisfaction was expressed as change in Diabetes Treatment Satisfaction Questionnaire (DTSQ) status version scores in the ONWARDS 6 trial and as mean DTSQ change version score in the QWINT‐5 trial; therefore, a meta‐analysis of results could not be performed.

## 4 DISCUSSION

The results of our meta‐analysis showed that, in people with type 1 diabetes, insulin icodec and insulin efsitora provide similar HbA1c reduction as compared with once‐daily basal insulins. The analysis of CGM metrics showed similar improvements from baseline in TIR and TAR with both once‐weekly and once‐daily basal insulins, thereby confirming comparable efficacy on glucose control. Interestingly, the glucose‐lowering effect of weekly insulin was obtained with savings in bolus insulin doses. Regarding safety, our analysis indicated that the use of weekly insulins compared to daily insulins in people with type 1 diabetes is not associated with increased body weight but can significantly increase the risk of both non‐severe and severe hypoglycaemic episodes.

Importantly, the calculated PI (1.254–5.082) suggests that the effect on severe, level 3 episodes is likely to remain significant also with future studies.

Hypoglycaemia rates in patients with type 1 diabetes treated with once‐weekly basal insulins are higher than those observed in patients with type 2 diabetes treated with the same regimen,^13^, ^26^ possibly due to differences in glycaemic variability, counterregulatory mechanisms, insulin sensitivity and the degree of endogenous insulin deficiency, all of which may contribute to distinct hypoglycaemia risks and patterns in these populations.

The increased risk of hypoglycaemia seen with once‐weekly versus once‐daily basal insulins in type 1 diabetes may plausibly reflect an interplay between pharmacological features of once‐weekly formulations and protocol‐driven titration approaches. In particular, the very long half‐life of weekly analogues may hinder effective down‐titration when insulin requirements decrease, while the relatively aggressive titration algorithms adopted in clinical trials (designed to achieve strict fasting glucose targets) may have amplified hypoglycaemia risk during the early treatment intensification phase. Moreover, the pharmacodynamic profile of once‐weekly basal insulins is not entirely flat, with molecule‐specific characteristics leading to periods of relatively higher activity followed by attenuation within the dosing interval.^10^

In line with this interpretation, in the original trials, most of level 3 episodes occurred during the titration period. It is also important to point out that level 3 hypoglycaemia tended to recur in a minority of participants (up to 4% for icodec and up to 10% for efsitora). Interestingly, in a post hoc analysis of the ONWARDS 6 trial, participants with CV ≤36% at weeks 0–2 reported lower rates of clinically significant or severe hypoglycaemic episodes from baseline to week 26 as compared with those with CV >36%, highlighting a potential role for CGM in the early identification of susceptible individuals.^27^ Taken together, these considerations suggest that drug‐related (insulin pharmacokinetics/pharmacodynamics), management‐related (titration strategies) and patient‐related (baseline glycaemic variability) factors all contribute to the increased risk of hypoglycaemia. Accordingly, more conservative titration protocols and careful patient selection, including early CGM‐based risk stratification, might help reduce hypoglycaemia occurrence in clinical practice.

Differently from what is observed in people with type 2 diabetes on a basal‐only regimen,^28^, ^29^ the impact of weekly insulins on treatment satisfaction in type 1 diabetes is unclear. It has been suggested that the perceived benefit of fewer basal insulin injections may be lower in individuals who also need to inject bolus insulin several times every day, as in adults with type 1 diabetes.^16^ While a meta‐analysis on measures of treatment satisfaction could not be performed for this specific outcome, we would like to highlight that the results of ONWARDS 6 and QWINT‐5 trials are conflicting, with the ONWARDS 6 reporting less favourable variations in DTSQ status version scores with icodec compared to degludec (ETD −1.09, 95% CI −1.85 to −0.34, *p* = 0.0044) and the QWINT‐5 reporting higher DTSQ change version scores for efsitora compared to degludec (14.4 ± 4.5 vs. 13.2 ± 5.2, *p* = 0.0081). Several factors may account for this discrepancy. First, ONWARDS 6 and QWINT‐5 differed in geographic coverage and participant characteristics. Indeed, ONWARDS 6^16^ included individuals from a wider range of countries and QWINT‐5 included people with slightly higher baseline HbA1c values.^14^ These differences could have influenced patient expectations and perceptions, even though baseline DTSQ scores appeared to be similar across the two studies. Moreover, the two studies investigated different molecules, each with molecule‐specific pharmacodynamic properties and titration algorithms, which may have conditioned patient perception of flexibility and convenience.^14^, ^16^ Further research, including evidence from real‐world studies, is needed to clarify this issue.

Our meta‐analysis is the first to evaluate the efficacy and safety of both insulin icodec and efsitora, focusing exclusively on people with type 1 diabetes and including all published RCTs. At present, there is only one additional study (NCT06807190) evaluating once‐weekly basal insulin therapy and registered in American (ClinicalTrials.gov) and European (EudraCT) clinical trial online databases; albeit, it is a single‐armed observational study and has not yet started recruitment. Of note, one recent meta‐analysis focused on type 1 diabetes,^30^ but did not include all available trials, while three previous meta‐analyses included individuals with both type 1 and type 2 diabetes; however, two focused solely on a single analogue,^31^, ^32^ and the third did not include the recent phase 3 trial QWINT‐5.^33^ By focusing exclusively on type 1 diabetes and integrating the most recent data, we provide more definitive conclusions on the efficacy and safety of these therapies, with a clear perspective on their potential role in clinical practice. In addition, our work adhered to a rigorous methodological approach, incorporating sophisticated statistical techniques to appropriately account for data heterogeneity and quality of included studies. Specifically, we employed PIs and the assessment of evidence certainty according to the GRADE methodology^18^ to clarify the clinical implications of our findings. Indeed, the robustness of our results is supported by the low heterogeneity of studies and moderate‐to‐high certainty of evidence for several outcomes.

It is known that insulin icodec and efsitora exhibit different biochemical structures half‐lives and titration algorithms.^34^, ^35^ However, all clinical endpoints included in our meta‐analysis were assessed at pharmacokinetic steady‐state, effectively ruling out differences in half‐life as a contributor to heterogeneity in results. Our meta‐analysis has some limitations that should be acknowledged. For some outcomes (i.e., DTSQ, TIR and TAR), it was necessary to impute some variability data, as detailed in the Methods section. Moreover, other CGM metrics could not be analysed due to the unavailability of variability data in most studies.

In very recent years, the advent of automated insulin delivery (AID) systems has led to unprecedented achievements in terms of TIR, TBR and glycaemic variability together with improved PROMs.^36^, ^37^ Accordingly, both guidelines and expert consensus recommend that all people with type 1 be offered an AID system over other treatment modalities.^38^, ^39^, ^40^ Nevertheless, multiple daily insulin injections therapy is still used by a significant proportion of people with type 1 diabetes around the world, and once‐weekly basal insulin may represent an opportunity to simplify insulin therapy in individuals without access to (or refusing) technological devices or exhibiting poor treatment adherence, with the additional aim of reducing the frequency of recurrent DKA episodes in the latter, provided that the above‐mentioned concerns on hypoglycaemia risk are resolved.

## 5 CONCLUSIONS

This meta‐analysis is the first to evaluate the efficacy and safety of once‐weekly basal insulins in the treatment of adults with type 1 diabetes, showing a similar glucose‐lowering effect with increased hypoglycaemia occurrence compared to once‐daily compounds.

## AUTHOR CONTRIBUTIONS

LDG, SDM, IC and FG contributed to the study conception and design. IC, LDG and SDM designed the statistical plan, performed the statistical search, collected the data and performed the analysis and the assessment of the certainty of evidence. SDM, LDG and IC gave the major contribution in writing the manuscript. LDG created the figures. LDG, IC and MC created the tables. LDG, SDM, IC, MC, GPS, AC, AN, SP, LL and FG revised the article and contributed to the discussion. FG supervised the project and finalized the manuscript. All authors read and approved the final manuscript. FG, LDG, SDM and IC accessed and verified the underlying data. FG is the guarantor of this work and, as such, had full access to all the data in the study and takes responsibility for the integrity of the data and the accuracy of the data analysis.

## CONFLICT OF INTEREST STATEMENT

AC: AstraZeneca, Eli Lilly, Novo Nordisk, Roche Diagnostics, Sanofi Aventis (honoraria). AN: AstraZeneca, Novo Nordisk and Sanofi Aventis (honoraria). FG: Eli Lilly, Roche Diabetes Care (grants); Eli Lilly, Novo Nordisk (consulting fees); AstraZeneca, Boehringer‐Ingelheim, Eli Lilly, Lifescan, Merck Sharp & Dohme, Medtronic, Novo Nordisk, Roche Diabetes Care, Sanofi Aventis (support for attending meetings or travels); AstraZeneca, Boehringer‐Ingelheim, Eli Lilly, Lifescan, Merck Sharp & Dohme, Medimmune, Medtronic, Novo Nordisk, Roche Diabetes Care, Sanofi Aventis (participation on Advisory Boards); EASD/EFSD, Società Italiana di Endocrinologia (SIE), Fo.Ri.SIE (unpaid leadership); AstraZeneca, Eli Lilly, Novo Nordisk, Sanofi Aventis (support for medical writing and statistical analysis). GPS: Eli Lilly, Novo Nordisk, Sanofi, Daichii Sankyo, Guidotti, Theramex, Farmitalia, Amryt (honoraria); Abbott (support for attending meetings or travels). IC: Eli Lilly, Novo Nordisk, Guidotti SpA, AstraZeneca (honoraria); Eli Lilly, Novo Nordisk, Abbott (support for attending meetings or travels). LDG: Eli Lilly, Roche Diabetes Care, MOVI SpA, Sanofi, Theras (honoraria); Abbott, Eli Lilly, Lusofarmaco, Novo Nordisk, Sanofi (support for attending meetings or travels); Eli Lilly (participation on Advisory Boards). LL: Abbott, AstraZeneca, Boehringer‐Ingelheim, Eli Lilly, Merck Sharp & Dohme, Medtronic, Menarini, MOVI SpA, Mundipharma, Novo Nordisk, Roche Diabetes Care, Sanofi Aventis, Terumo (honoraria); Abbott, AstraZeneca, Boehringer‐Ingelheim, Eli Lilly Italia, Medtronic, MOVI SpA, Novo Nordisk, Roche Diabetes Care, Sanofi Aventis, Terumo (participation on Advisory Boards). MC: Eli Lilly, Lusofarmaco, Novo Nordisk, Sanofi (support for attending meetings or travels). SDM: Ascensia, MOVI SpA, Roche Diabetes Care (honoraria); Ascensia, MOVI SpA, Roche Diabetes Care (participation on Advisory Boards). SP: AstraZeneca, Eli Lilly, Novo Nordisk, Sanofi Aventis (honoraria).

## ETHICS STATEMENT

Analyses were performed on data extracted from published papers. Patient consent for publication was not required.

## Supporting information


**Data S1.** Supporting Information.

## Data Availability

All data relevant to the study are included in the article or uploaded as Supporting Information. Statistical code and data set: available on reasonable request from Prof. Francesco Giorgino (francesco.giorgino@uniba.it).
