# Supplementary material for: Efficacy and safety of once‐weekly basal insulin therapy in people with type 1 diabetes: A systematic review and meta‐analysis
Source: Diabetes Obes Metab. 2025 Oct 6;28(1):210–20. doi: 10.1111/dom.70176 (PMC12673469; doi:10.1111/dom.70176)
Supplement: Supplementary file 1 — Data S1. Supporting Information. [file DOM-28-210-s001.docx]

**Supplementary Appendix**

Efficacy and safety of once-weekly basal insulin therapy in people with type 1 diabetes: a systematic review and meta-analysis

**Contents**

[**Supplement 1. Search strategy 3**](#_jxfuwkmc0slh)

[**Supplement 2. Characteristics of studies and patients’ baseline features 5**](#_dwvs5qbilsma)

[**Supplement 3. Risk of bias assessment of included trials for each outcome 9**](#_xd9nsmz4wlal)

[**Supplement 4. Funnel plots 11**](#_m9z5t38mty1u)

[**Supplement 5. Forest plots 18**](#_71so063tj35p)

[*5.1 HbA1c 18*](#_df1pd2l2dldk)

[*5.2 Fasting plasma glucose 18*](#_rjqv96q31vd)

[*5.3 Body weight 19*](#_gehh00u1ptlr)

[*5.4 Time in range (70-180 mg/dL) 19*](#_byqjz44dv1tp)

[*5.5 Time above range (>180 mg/dL) 20*](#_9bz3k2d1sv18)

[*5.6 Time above range level 1 (181-250 mg/dL) 21*](#_fprz861fxhh4)

[*5.7 Time above range level 2 (>250 mg/dL) 21*](#_3qxjmxwbgyp9)

[*5.8 Time below range level 1 (54-69 mg/dL) 21*](#_ggjsss6ixvq9)

[*5.9 Time below range level 2 (<54 mg/dL) 21*](#_1halxv2i3t85)

[*5.10 Level 1 hypoglycemic events 22*](#_yxiteyw050hm)

[*5.11 Level 2 hypoglycemic events 23*](#_g66kdx1sapu7)

[*5.12 Level 3 hypoglycemic events 24*](#_r2fs6r8bpgu5)

[*5.13 Total insulin dose 25*](#_imk537fbo5ix)

[*5.14 Basal insulin dose 25*](#_3zj7osjbz41a)

[*5.15 Bolus insulin dose 26*](#_yc1akuaq0kms)

[**Supplement 6. Summary of findings 27**](#_a6oyws49hei6)

[**Supplement 7. Other severe adverse events 28**](#_ub5h34w4lfa8)

[**References 29**](#_epyz7440477)

##

## Supplement 1. Search strategy

**MEDLINE (via Ovid):**

1. diabet*.mp.

2. Diabetes Mellitus/

3. exp Diabetes Mellitus Type 1/

4. ((diabetes or "diabetes mellitus" or diabetic*) and (type 1 or "type I" or "type i" or "insulin dependent" or latent or autoimmune)).mp. [mp=ti, ab, tx, ct, ot, bt, hw, id, cc, nm, fx, kf, ox, px, rx, ui, sy, ux, mx]

5. "IDDM".mp.

6. "LADA".mp.

7. or/1-6

8. ("weekly insulin" OR "once-weekly insulin" OR "weekly basal insulin" OR "icodec" OR "weekly icodec" OR "efsitora" OR "weekly efsitora").mp.

9. randomized controlled trial.pt.

10. controlled clinical trial.pt.

11. pragmatic clinical trial.pt.

12. randomized.ab.

13. randomised.ab.

14. randomly.ab.

15. trial.ti.

16. or/9-15

17. and/7, 8, 16

18. remove duplicates from 17

<https://ovidsp.ovid.com/ovidweb.cgi?T=JS&NEWS=N&PAGE=main&SHAREDSEARCHID=5cPXSkZ46s6XV2bmJLMqcWadxCJ8lzlKc9dLVyHwvXoTyri7SWwpigdc6nLInvFfc>

**Web of science:**

(TS=(diabet* OR "Diabetes Mellitus" OR "Diabetes Mellitus Type 1" OR ((diabetes OR "diabetes mellitus" OR diabetic*) AND (type 1 OR "type I" OR "type i" OR "insulin dependent" OR latent OR autoimmune)) OR "IDDM" OR "LADA"))

AND

(TS=("weekly insulin" OR "once-weekly insulin" OR "weekly basal insulin" OR "icodec" OR "weekly icodec" OR "efsitora" OR "weekly efsitora"))

AND

(TS=("randomized controlled trial" OR "controlled clinical trial" OR "pragmatic clinical trial" OR randomized OR randomised OR randomly OR trial))

**CENTRAL:**

(diabet* OR "Diabetes Mellitus" OR "Diabetes Mellitus Type 1" OR ((diabetes OR "diabetes mellitus" OR diabetic*) AND (type 1 OR "type I" OR "type i" OR "insulin dependent" OR latent OR autoimmune)) OR "IDDM" OR "LADA")

AND

("weekly insulin" OR "once-weekly insulin" OR "weekly basal insulin" OR "icodec" OR "weekly icodec" OR "efsitora" OR "weekly efsitora")

AND

("randomized controlled trial" OR "controlled clinical trial" OR "pragmatic clinical trial" OR randomized OR randomised OR randomly OR trial)

##

## Supplement 2. Characteristics of studies and patients’ baseline features

|  | **Kazda C.M., 2023** [(1)](https://paperpile.com/c/BO6zg2/BU3t8) | **Russell-Jones D., 2023**  **(ONWARDS 6)** [(2)](https://paperpile.com/c/BO6zg2/im1iT) | **Hövelmann U., 2024** [(3)](https://paperpile.com/c/BO6zg2/vM4bi) | **Bergenstal R.M., 2024**  **(QWINT-5)** [(4)](https://paperpile.com/c/BO6zg2/Nzqyn) | **Eto T., 2024** [(5)](https://paperpile.com/c/BO6zg2/he2xf) |
| --- | --- | --- | --- | --- | --- |
| **Clinical trial number** | NCT04450407 | NCT04848480 | NCT03723772 | NCT05463744 | NCT03766854 |
| **Population** | T1D on basal-bolus regimen | T1D on basal-bolus regimen | T1D on basal-bolus regimen or continuous subcutaneous insulin infusion | T1D on basal-bolus regimen | T1D on basal-bolus regimen or continuous subcutaneous insulin infusion |
| **Number of sites** | 49 | 99 | 1 | 82 | 1 |
| **Study design** | Multicenter, randomized, parallel, open-label, comparator-controlled, phase 2 study | Randomised, multicentre,  open-label, active-controlled, parallel-group, treat-to-  target, phase 3a trial | Randomized, single-centre, open-label, two-period crossover trial | Randomised, multicentre, parallel-design, open-label, treat-to-target non-inferiority study, phase 3 trial | Randomized, single-centre, open-label, two-period crossover trial |
| **Screening periods** | 2 weeks | 2 weeks | 2-70 days | 3 weeks | 2-70 days |
| **Treatment duration** | 26 weeks | 52 weeks (26 weeks main phase, 26 weeks safety extension phase) | *Treatment period randomization*:  8 weeks (icodec),  14 days (glargine U100);  *Buffer period*:  35-49 days (icodec),  2-15 days (glargine U100)  *Treatment period cross-over*:  8 weeks (icodec),  14 days (glargine U100) | 52 weeks treatment period | *Treatment period*:  8 weeks (icodec),  14 days (glargine U100);  *Buffer period*:  35-49 days (icodec),  2-15 days (glargine U100) |
| **Follow-up period** | 5 weeks | 5 weeks | 39-45 days (icodec)  5-11 days (glargine U100) | 5 weeks | 39-45 days (icodec)  5-11 days (glargine U100) |
| **Inclusion criteria** | T1D adults (≥18 years), HbA_1c_ 5.6-9.5% (38-80 mmol/moL), fasting C-peptide ≤0.30 nmol/L, BMI ≤35 kg/m^2^, with no substantial weight change (≥5%)  in the past 3 months. Previously treated a stable regimen of multiple daily injections for at least 3 months | T1D adults (≥18 years), HbA_1c_ <10% (86 mmol/mol), previously treated with multiple daily insulin injections (basal-bolus insulin analogue regimens) for at least 1 year | Men and women aged 18-64 years, with T1D for ≥12 months, HbA_1c_ ≤ 9% (≤75 mmol/moL), fasting C-peptide <0.3 nmol/L, BMI 18.5-29 kg/m^2^, previously treated with multiple daily insulin injections or continuous subcutaneous insulin infusion with daily basal dose ≥0.2 U/kg/day | T1D adults (≥18 years), HbA_1c_ 7-10% (53–86 mmol/moL), previously treated with a regimen of basal and bolus insulin analogue multiple daily injection therapy for at least 90 days before screening | Japanese patients aged 20-64 years, with T1D for ≥1 year, HbA_1c_ ≤ 9% (≤75 mmol/moL), fasting C-peptide <0.3 nmol/L, BMI 18.5-28 kg/m^2^ |
| **Intervention** | Once-weekly efsitora | Once-weekly icodec | Once-weekly icodec | Once-weekly efsitora | Once-weekly icodec |
| **Comparator** | Once-daily degludec | Once-daily degludec | Once-daily glargine U100 | Once-daily degludec | Once-daily glargine U100 |
| **Sample size** | Efsitora: efficacy population (n = 123), algorithm 2 population (n = 16), pooled safety population (n = 139)  Degludec: 126 | Icodec: 290  Degludec: 292 | Icodec: 66  Glargine U100: 66 | Efsitora: 343  Degludec: 349 | Icodec: 24  Glargine U100: 24 |
| **Age, years [mean (SD)]** | Efsitora: 45.5 (15.3)  Degludec: 47.4 (13.7) | Icodec: 44.1 (14.1)  Degludec: 44.3 (14.1) | 42.9 (12.8) | Efsitora: 44.4 (14.2)  Degludec: 43.6 (14) | 38.3 (9.8) |
| **Male [n (%)]** | Efsitora: 86 (61.9)  Degludec: 78 (61.9) | Icodec: 165 (57)  Degludec: 172 (59) | 56 (84.8) | Efsitora: 193 (56)  Degludec: 191 (55) | 14 (58.3) |
| **Diabetes duration, years [mean (SD), or median (IQR)]** | Efsitora: 22.0 (13.1)  Degludec: 22.3 (13.9) | Icodec: 20 (13.2)  Degludec: 19 (12.9) | 23.1 (10.5) | Efsitora: 17.1 (10.1-26.9)  Degludec: 17.9 (11.1-26.9) | 13.0 (10.9) |
| **BMI, kg/m^2^**  **[mean (SD)]** | Efsitora: 27.5 (4.0)  Degludec: 27.2 (4.1) | Icodec: 26.8 (5.0)  Degludec: 26.2 (4.5) | 26.1 (2.1) | Efsitora: 26.5 (4.0)  Degludec: 25.9 (4.1) | 22.7 (2.5) |
| **Baseline FPG, mg/dL [mean (SD), median (IQR)]** | Efsitora: 165.4 (67.9)  Degludec: 159.3 (67.1) | Icodec: 179 (74)  Degludec: 172 (72) | - | Efsitora: 153.6 (113-205)  Degludec: 160 (118-214) | - |
| **Baseline HbA_1c_, %**  **[mean (SD)]** | Efsitora: 7.5 (0.8)  Degludec: 7.5 (0.9) | Icodec: 7.59 (0.96)  Degludec: 7.63 (0.93) | 7.2 (0.7) | Efsitora: 7.88 (0.75)  Degludec: 7.94 (0.72) | 7.7 (0.6) |
| **Post-treatment HbA_1c_, % [mean, or mean (SD)]** | Efsitora: 7.5  Degludec: 7.33 | Icodec: 7.15  Degludec: 7.1 | - | Efsitora: 7.51 (0.39)  Degludec: 7.47 (0.44) | - |
| **Total insulin dose, U/week; U/day** | Only represented graphically in Supplemental Figure 5. Insulin Dose | Icodec: 310 (~44)  Degludec: 329 (~47) | Icodec: 1.91 (± 0.44)  Glargine U100: 0.27 (± 0.06) | Efsitora: 355.7 (50.8)  Degludec: 391.6 (55.9) | - |
| **Outcomes** | *Primary outcome:*  HbA_1c_  *Secondary outcomes:*  FPG, glycemic variability | *Primary outcome:*  HbA_1c_  *Secondary outcomes:*  FPG, TIR, TBR, TAR, hypoglycemic events, change in body weight, insulin doses, diabetes treatment satisfaction | *Primary outcome:*  Total icodec exposure at steady state (AUCτ,SS) | *Primary outcome:*  HbA_1c_  *Secondary outcomes:*  Hypoglycemic events, TIR, TBR, insulin doses, diabetes treatment satisfaction | *Primary outcome:*  to assess the pharmacological characteristics of icodec in Japanese individuals and relate the results to those in White individuals |

T1D: Type 1 Diabetes; BMI: Body Mass Index; SD: Standard Deviation; IQR: Interquartile Range; FPG: Fasting Plasma Glucose; TIR: Time in Range, TBR: Time Below Range; TAR: Time Above Range. For Kazda C.M. 2023, age, gender, diabetes duration, BMI, baseline FPG, baseline HbA1c, post-treatment HbA1c, and total insulin dose refer to the pooled safety population. - not reported.

##

## Supplement 3. Risk of bias assessment of included trials for each outcome

##
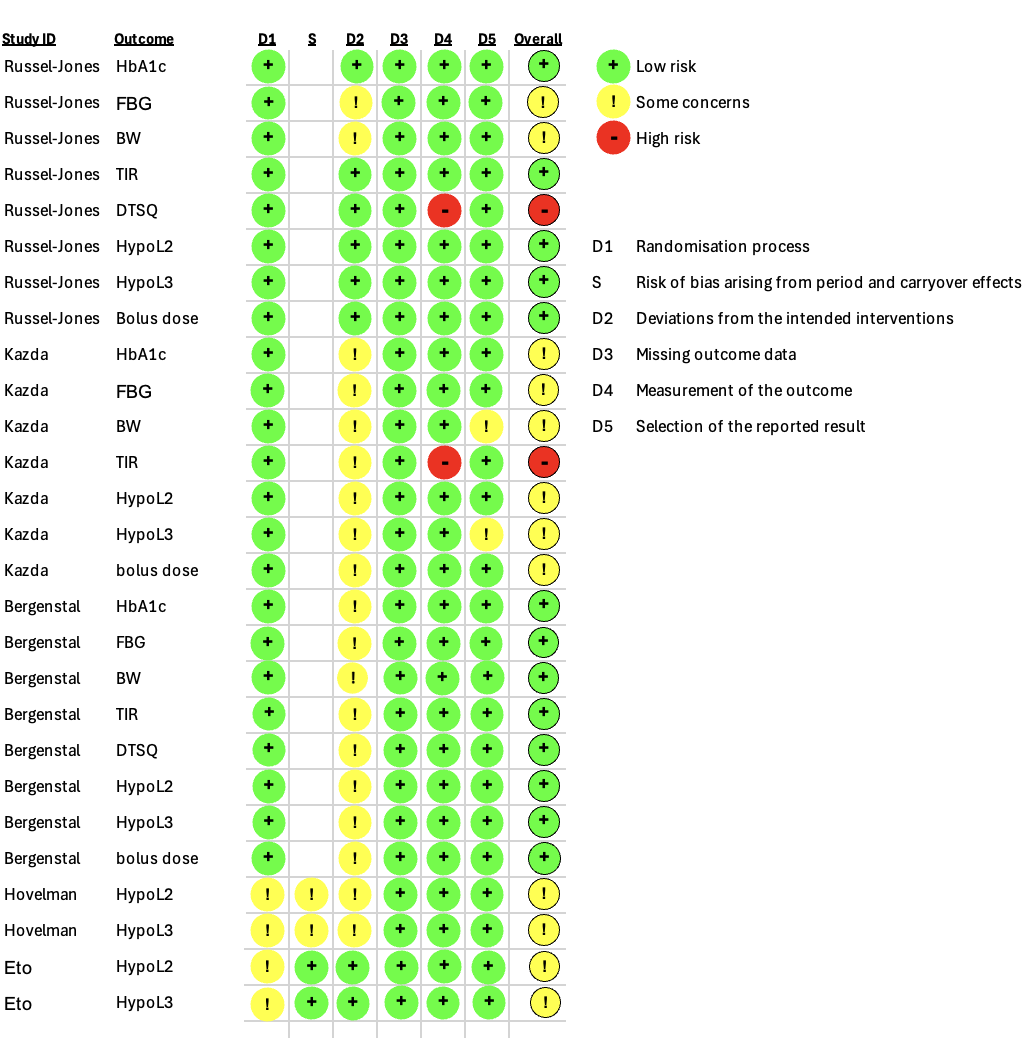


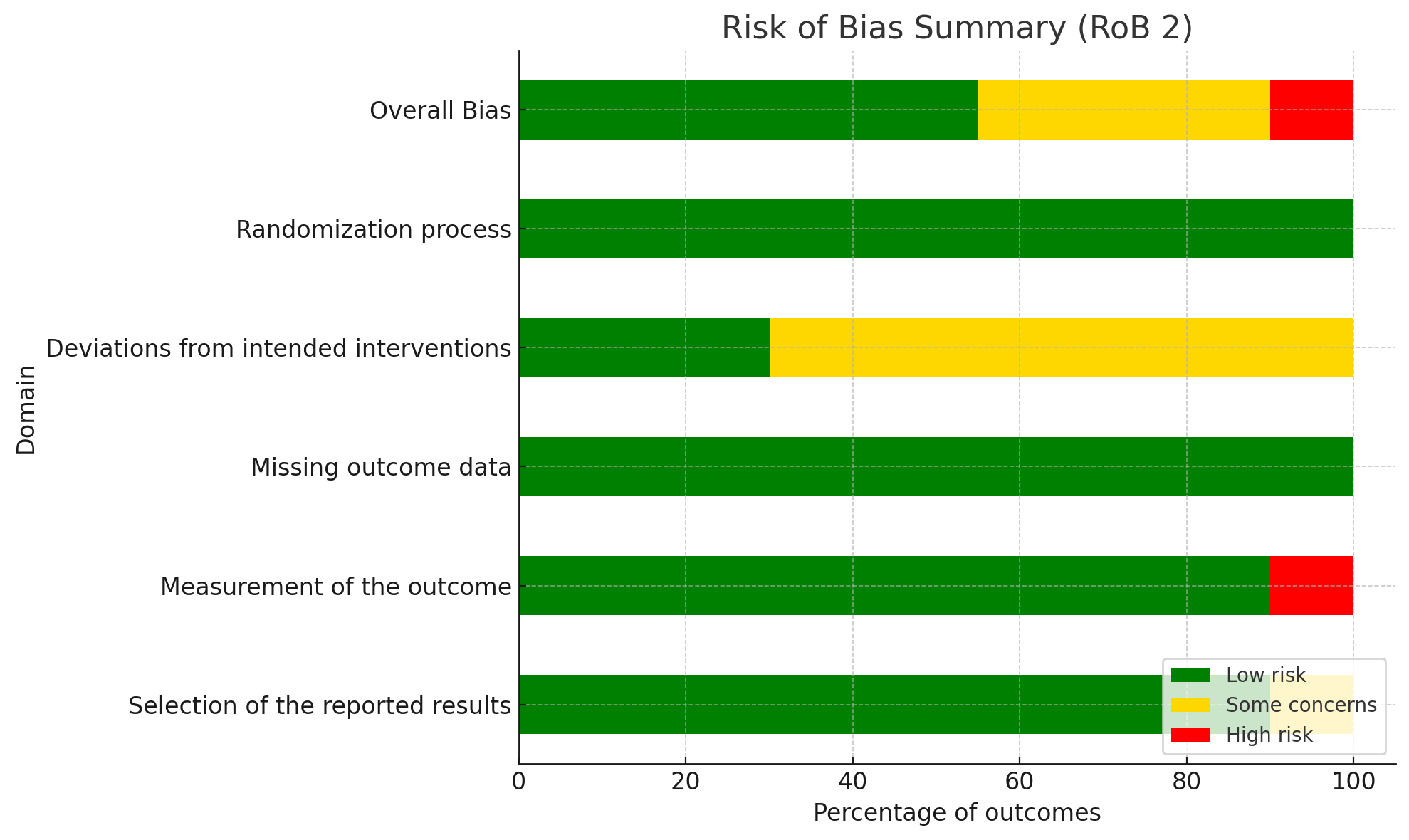

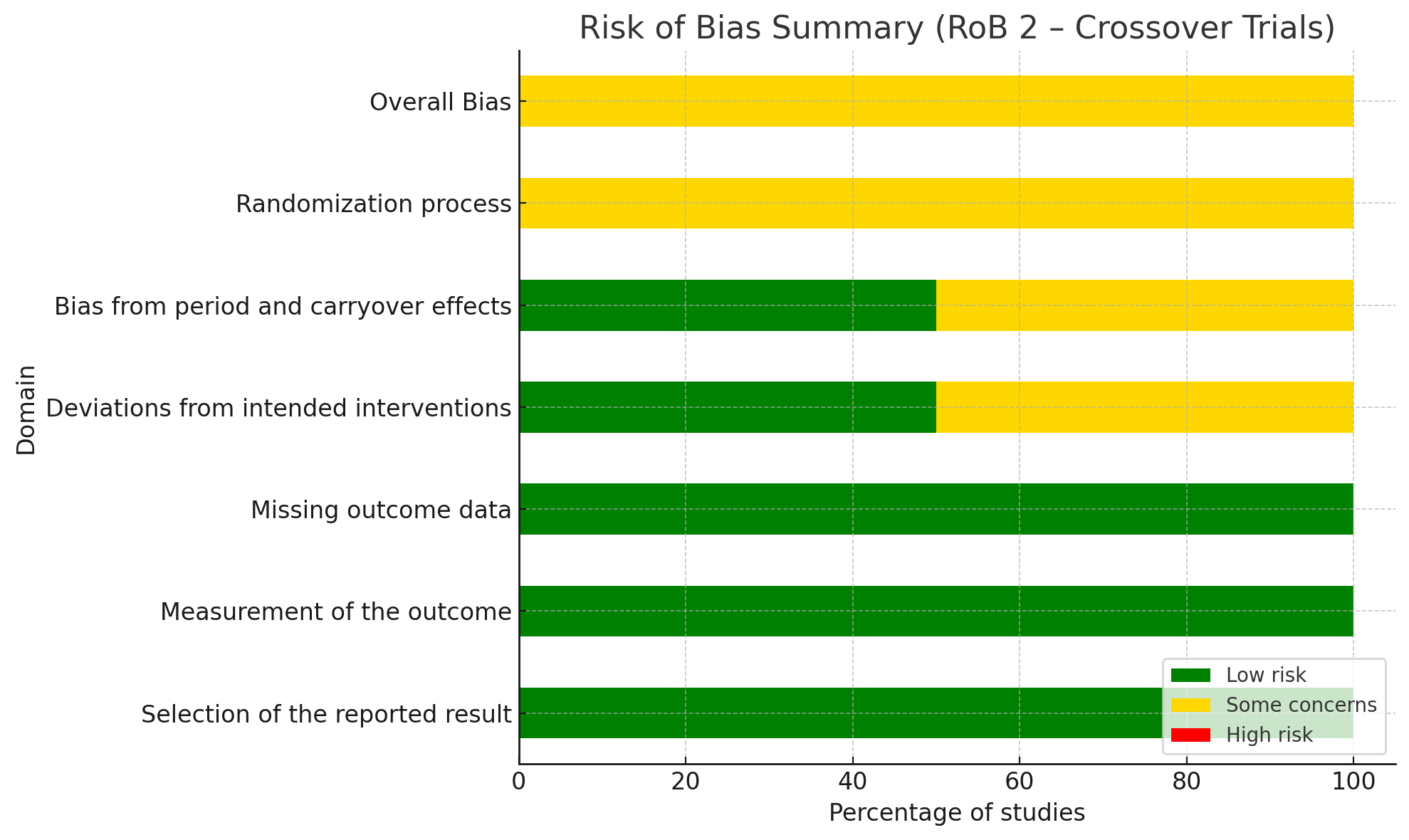


## Supplement 4. Funnel plots

##

##
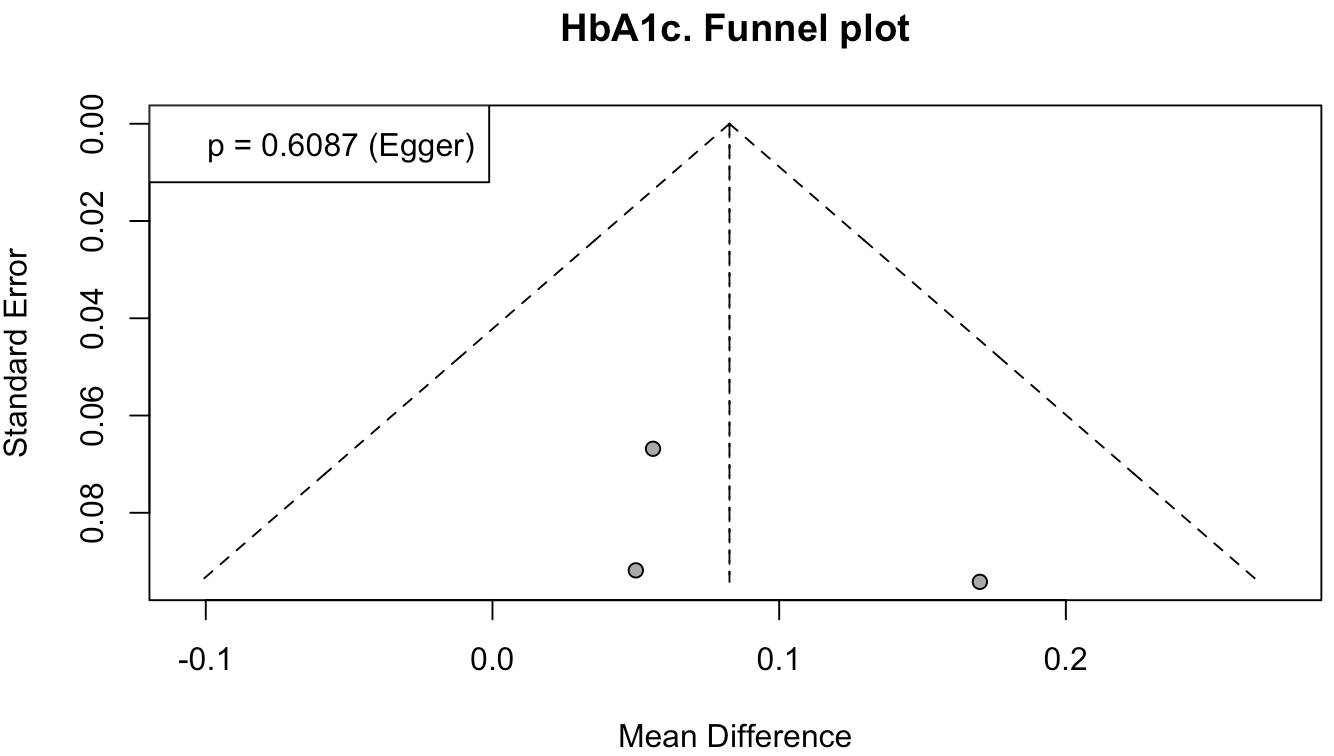


##
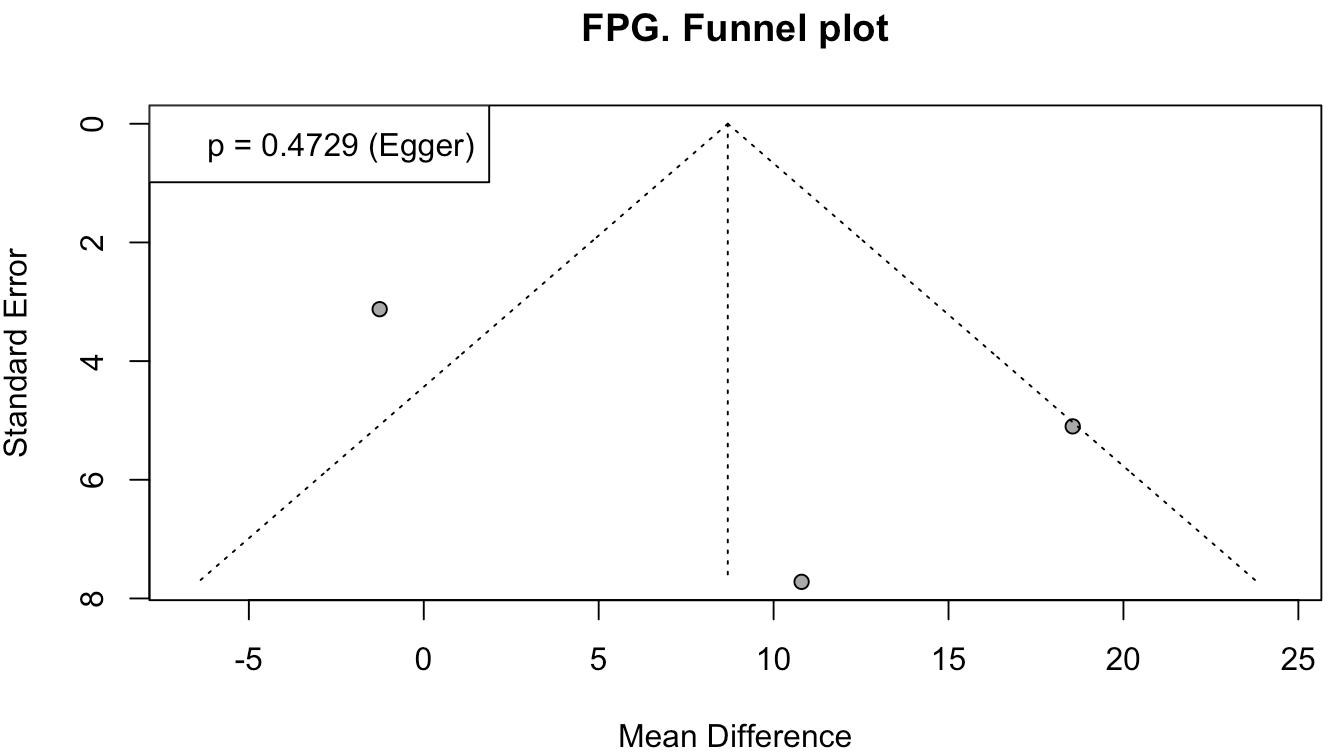


##
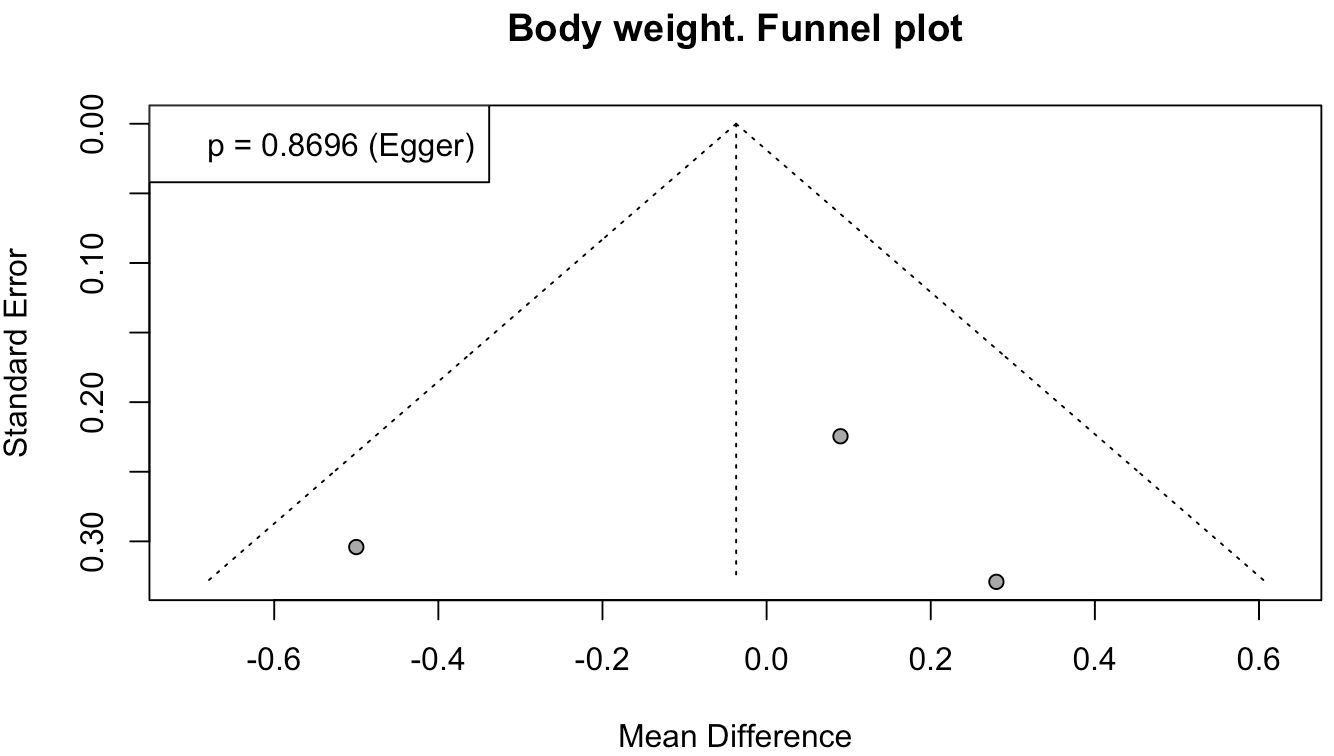

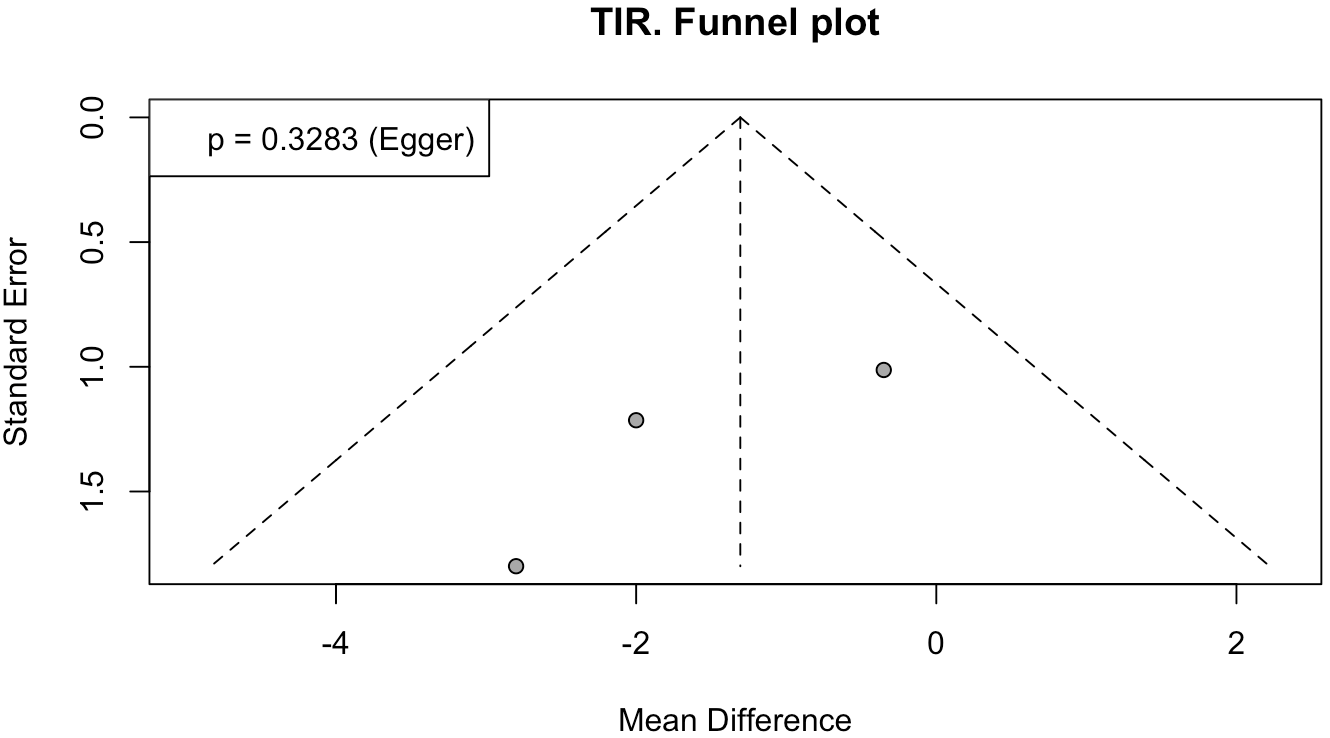

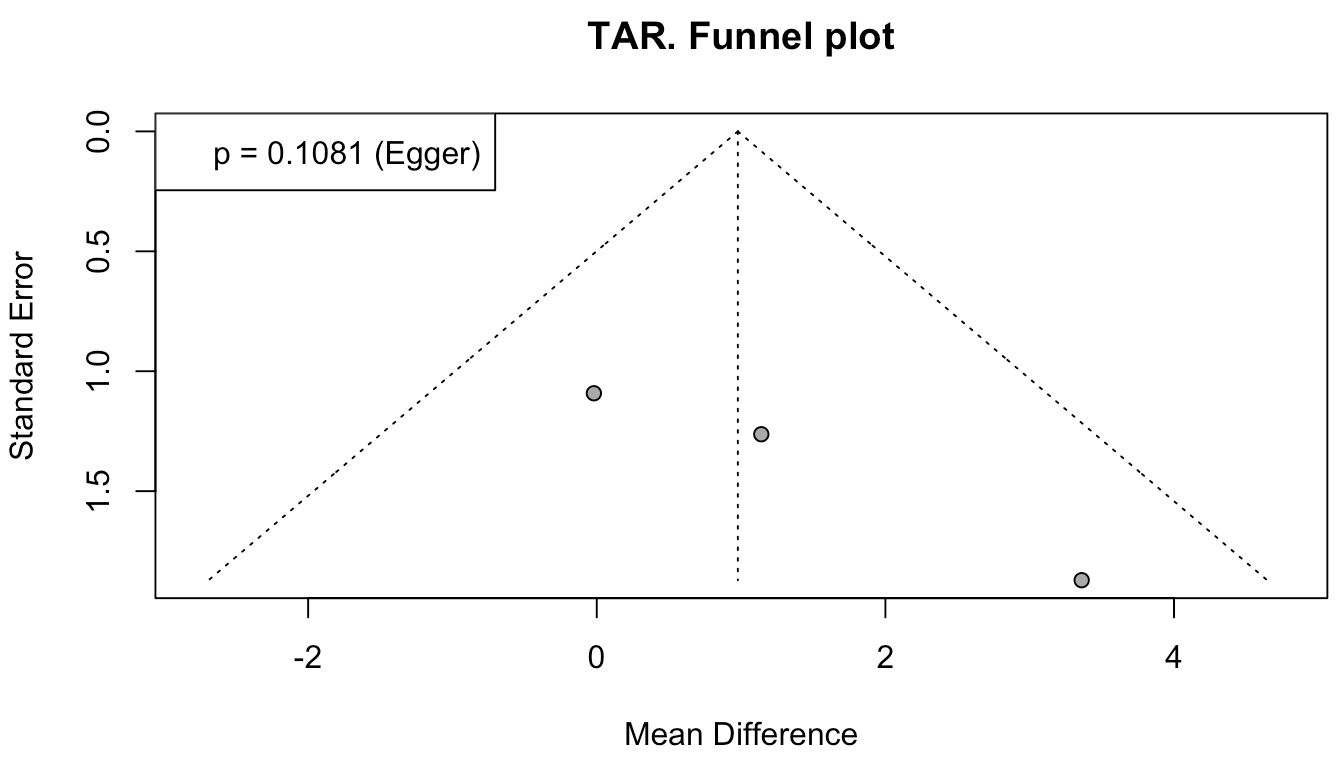

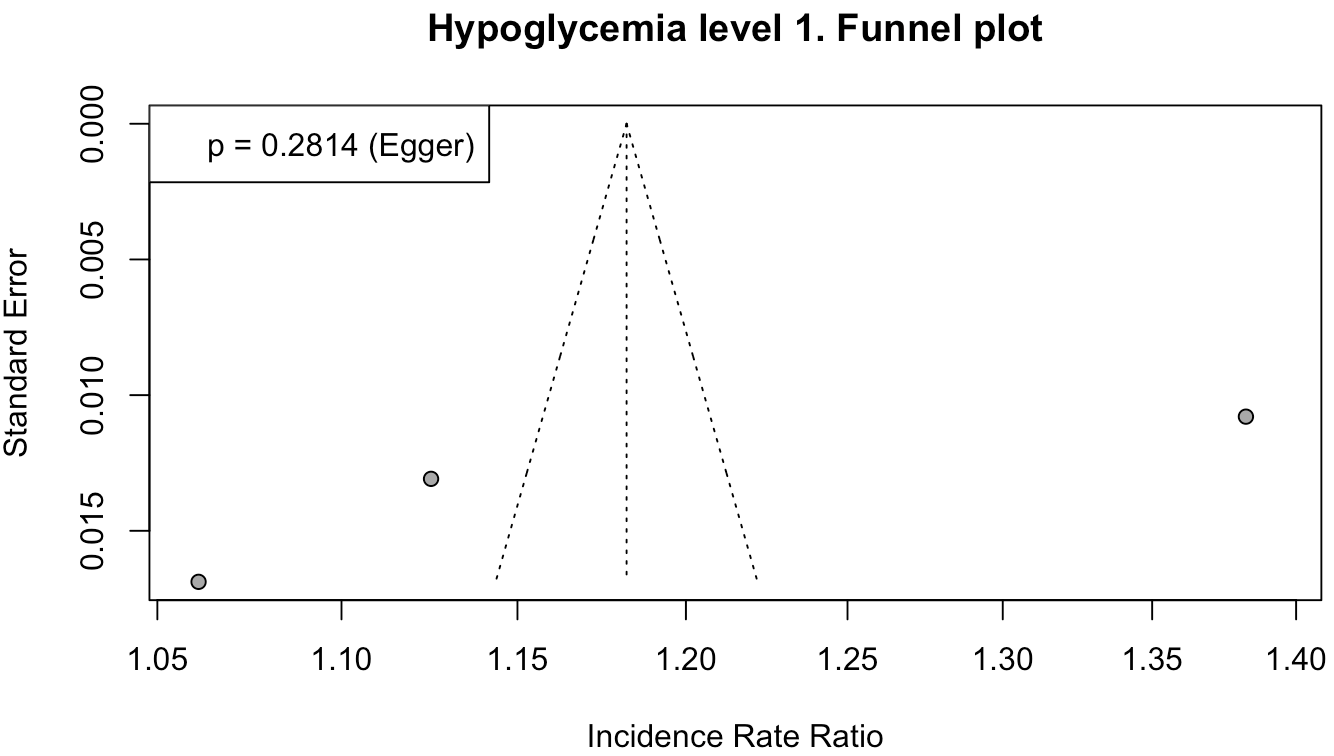

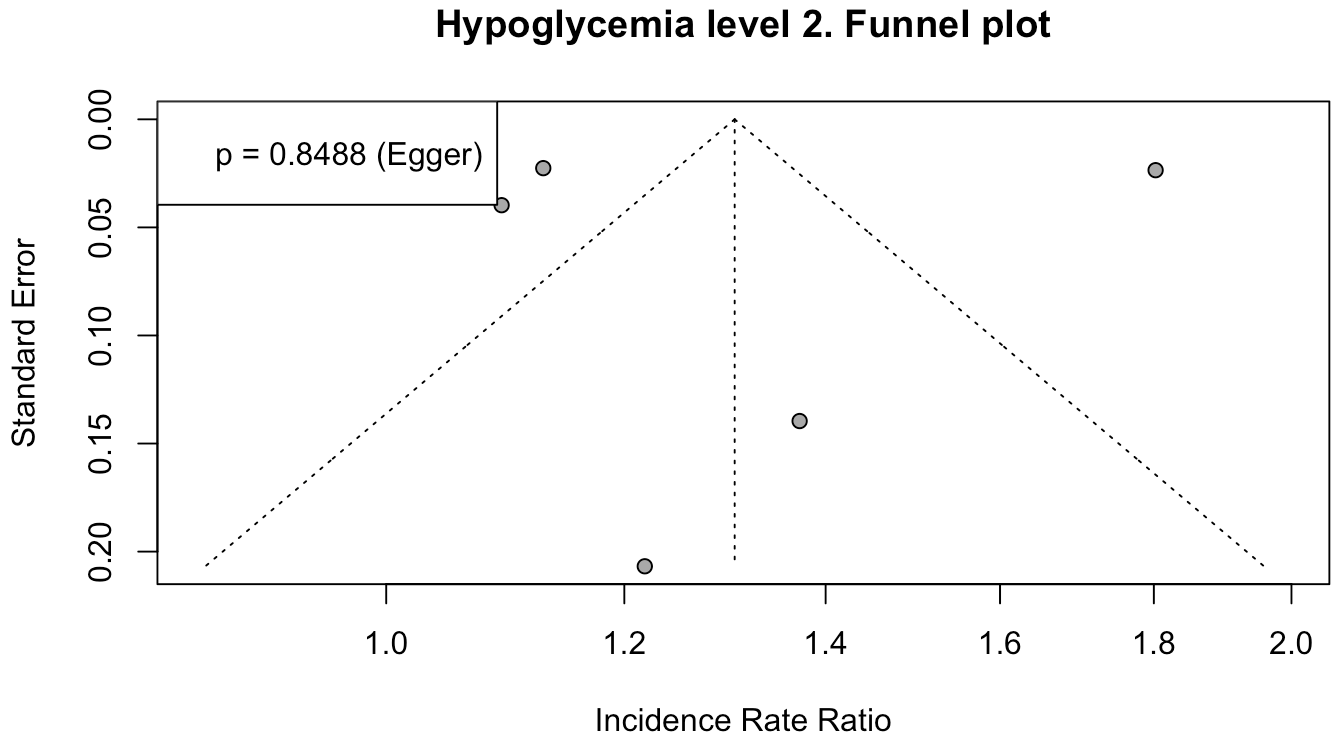

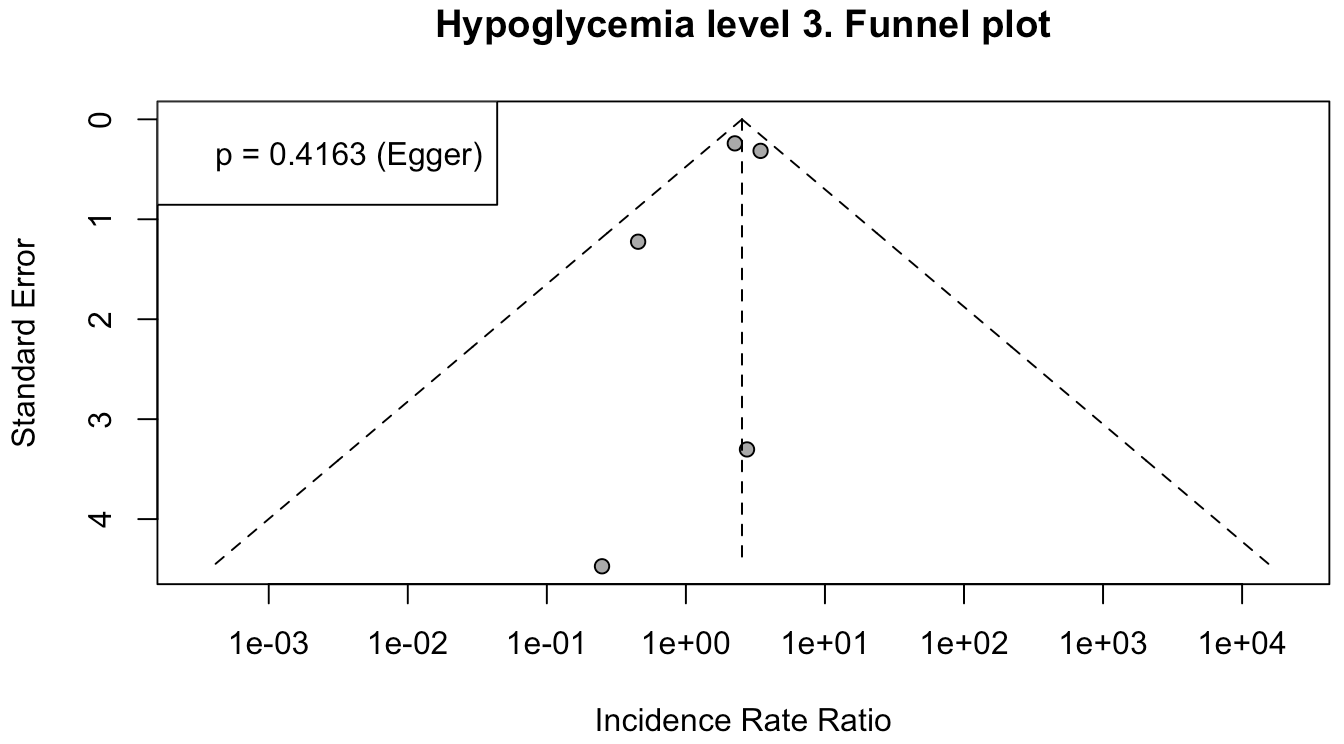

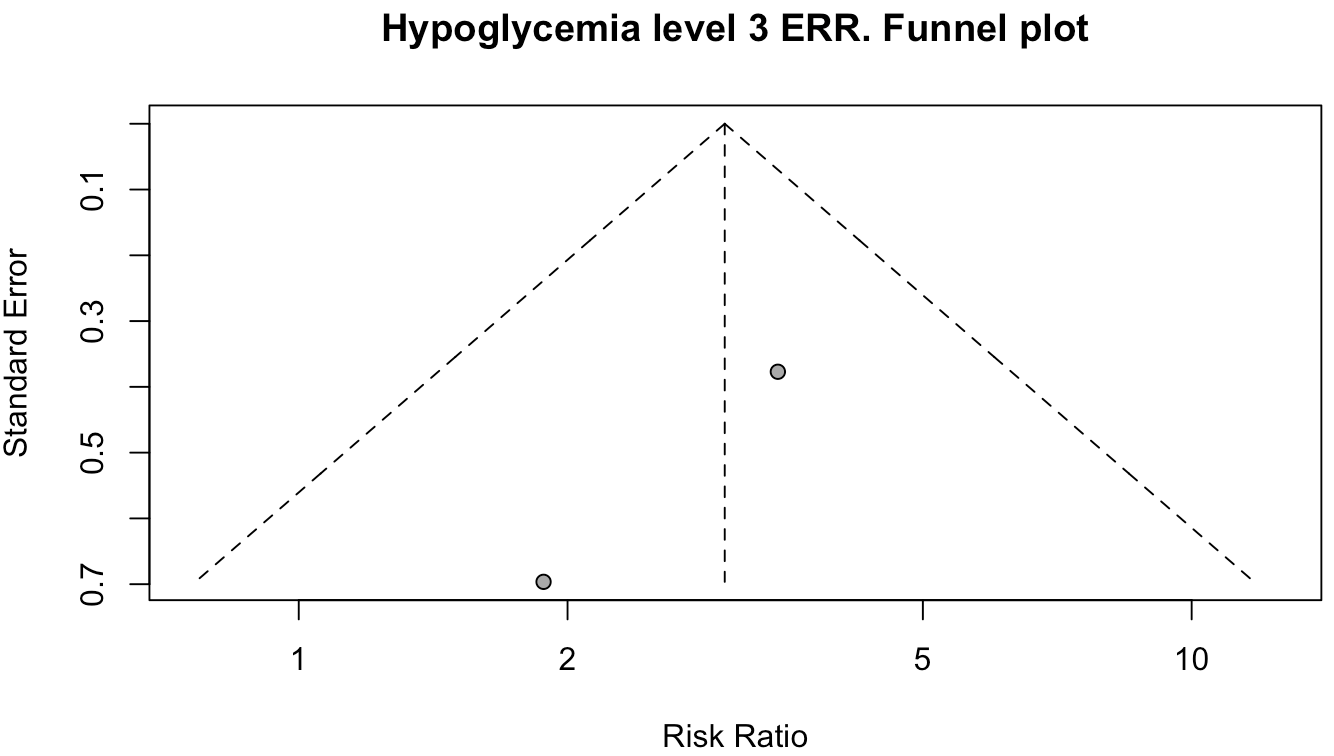

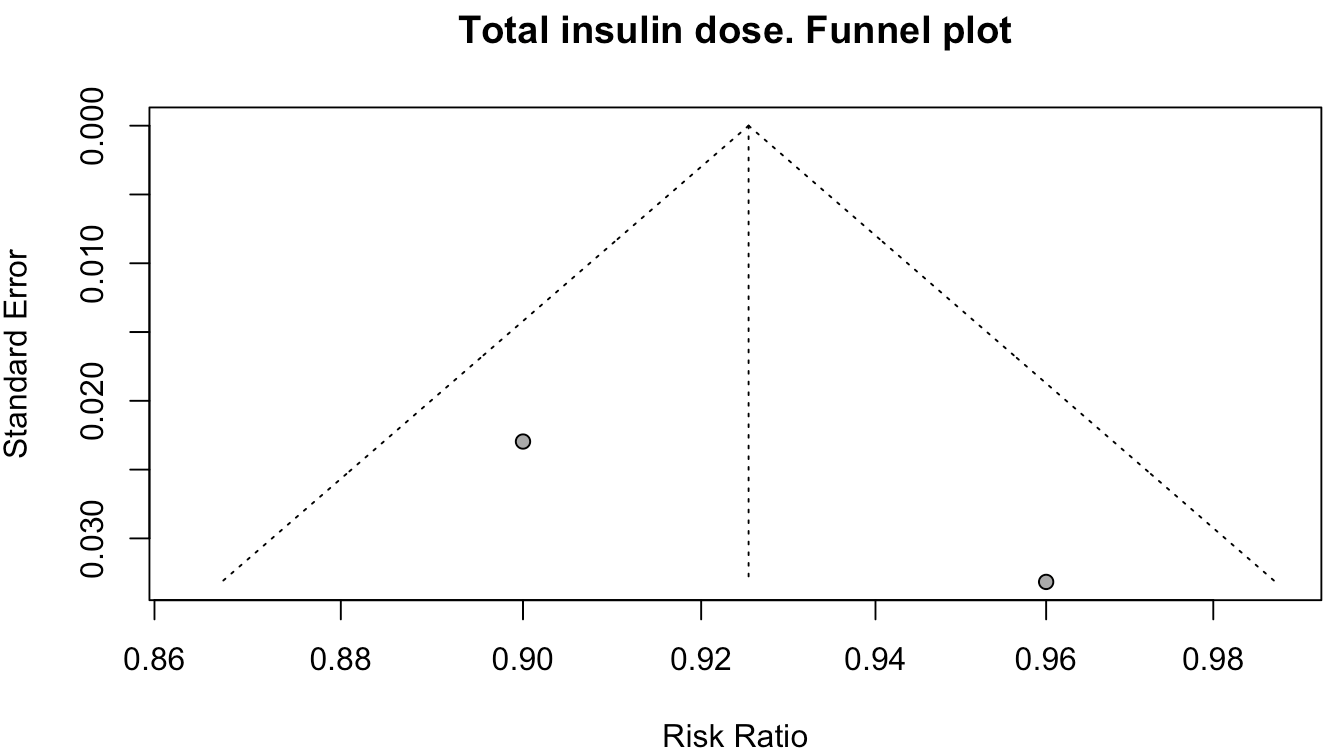

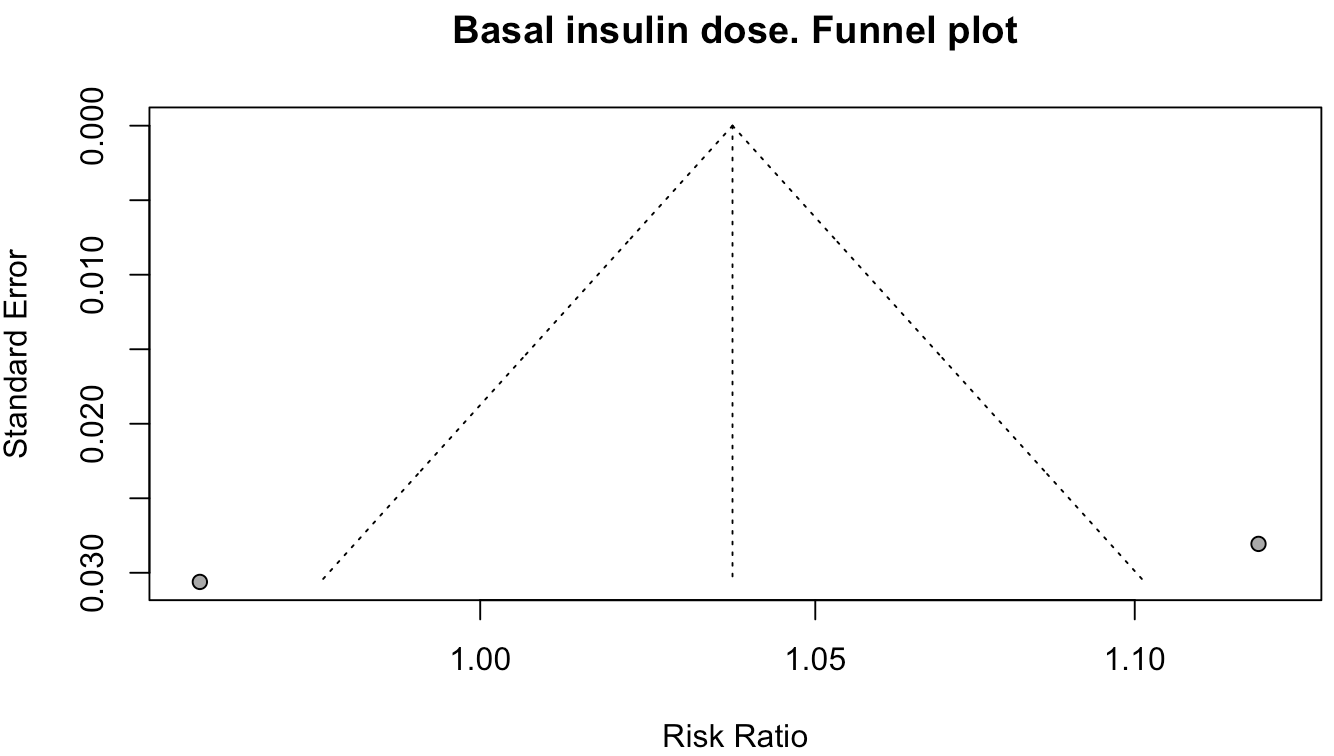

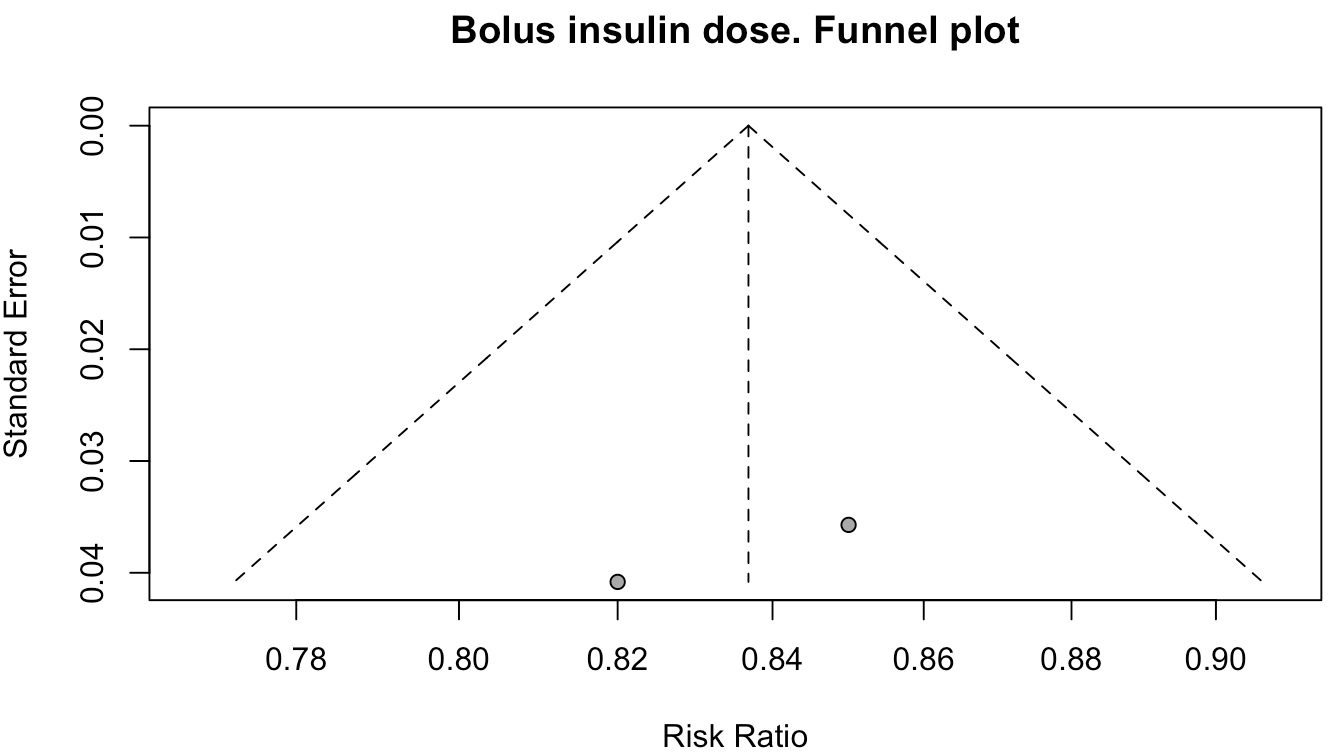


##
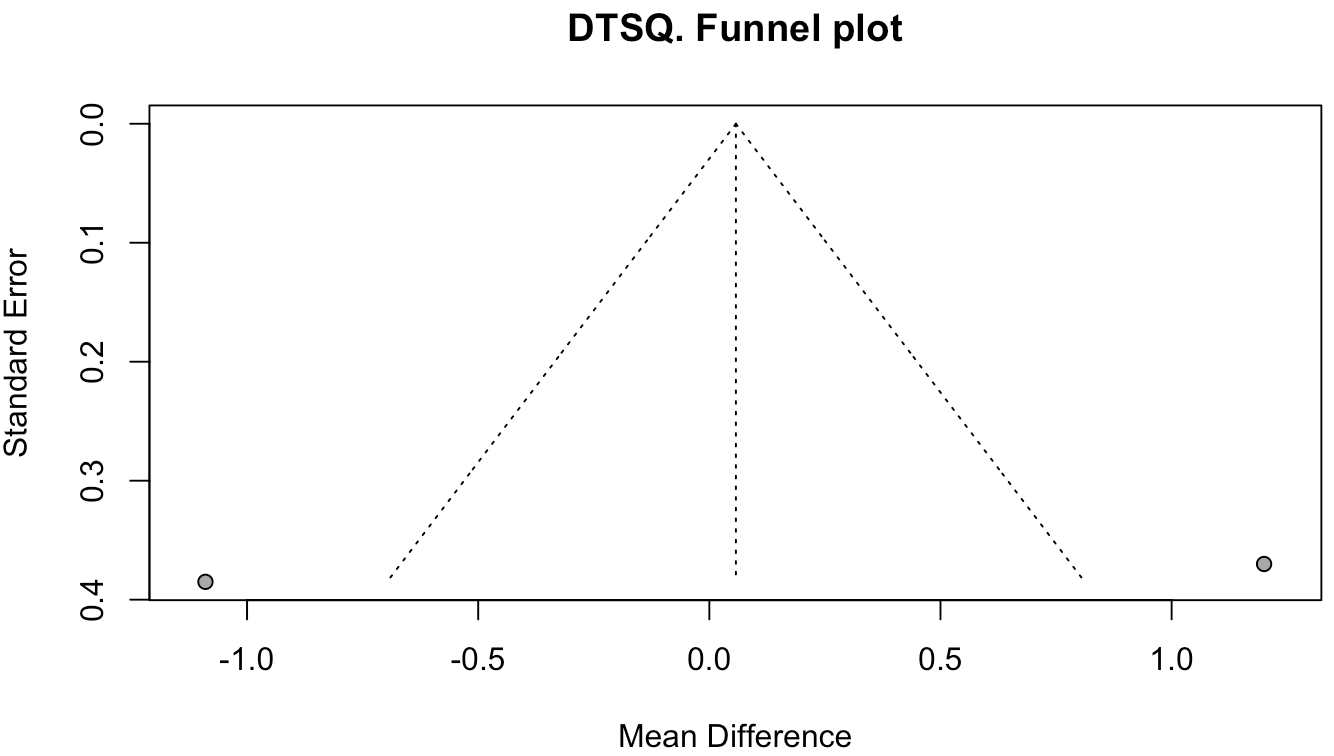


## Supplement 5. Forest plots

##

### 5.1 HbA1c

###
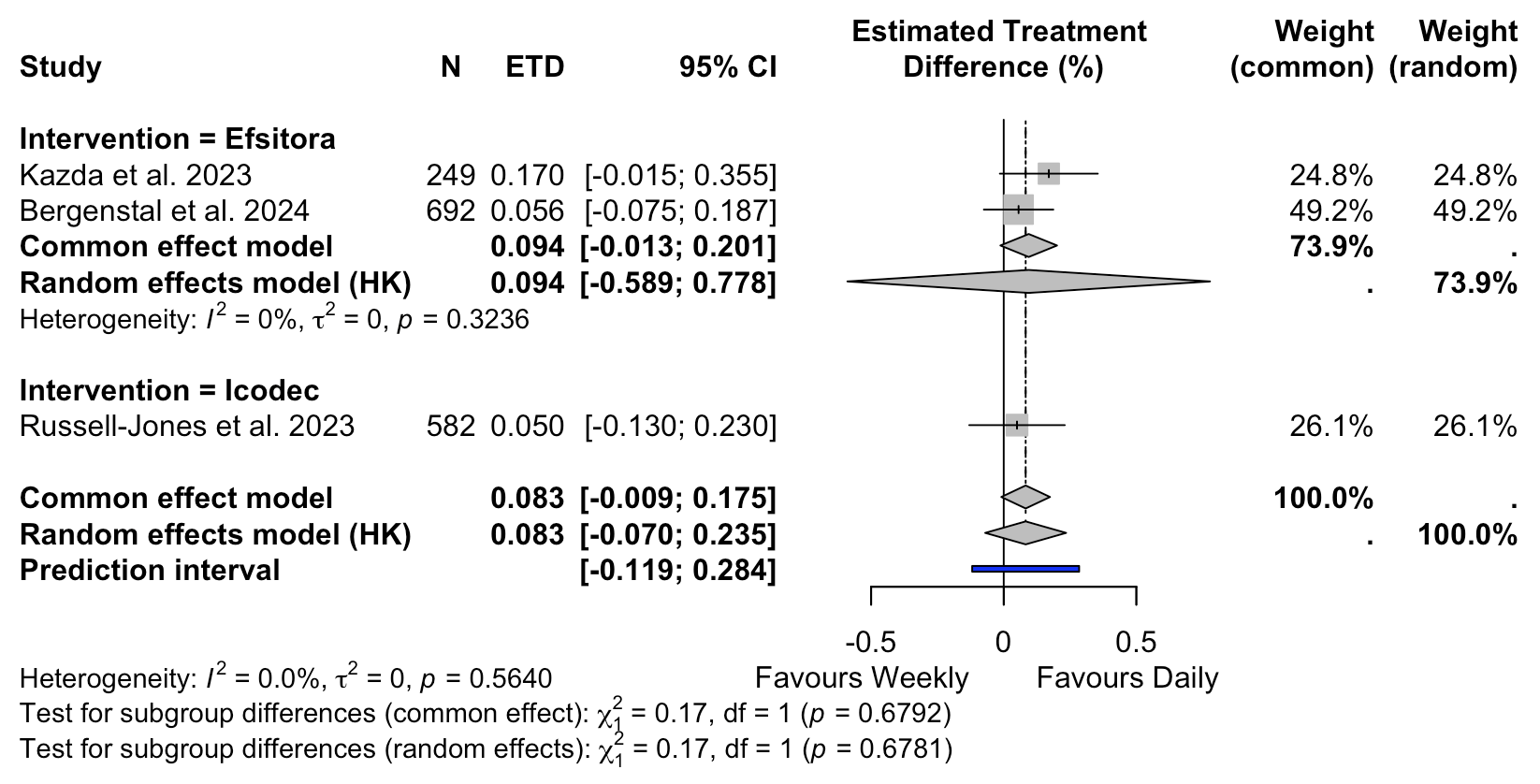


###

###

### 5.2 Fasting plasma glucose

###

###
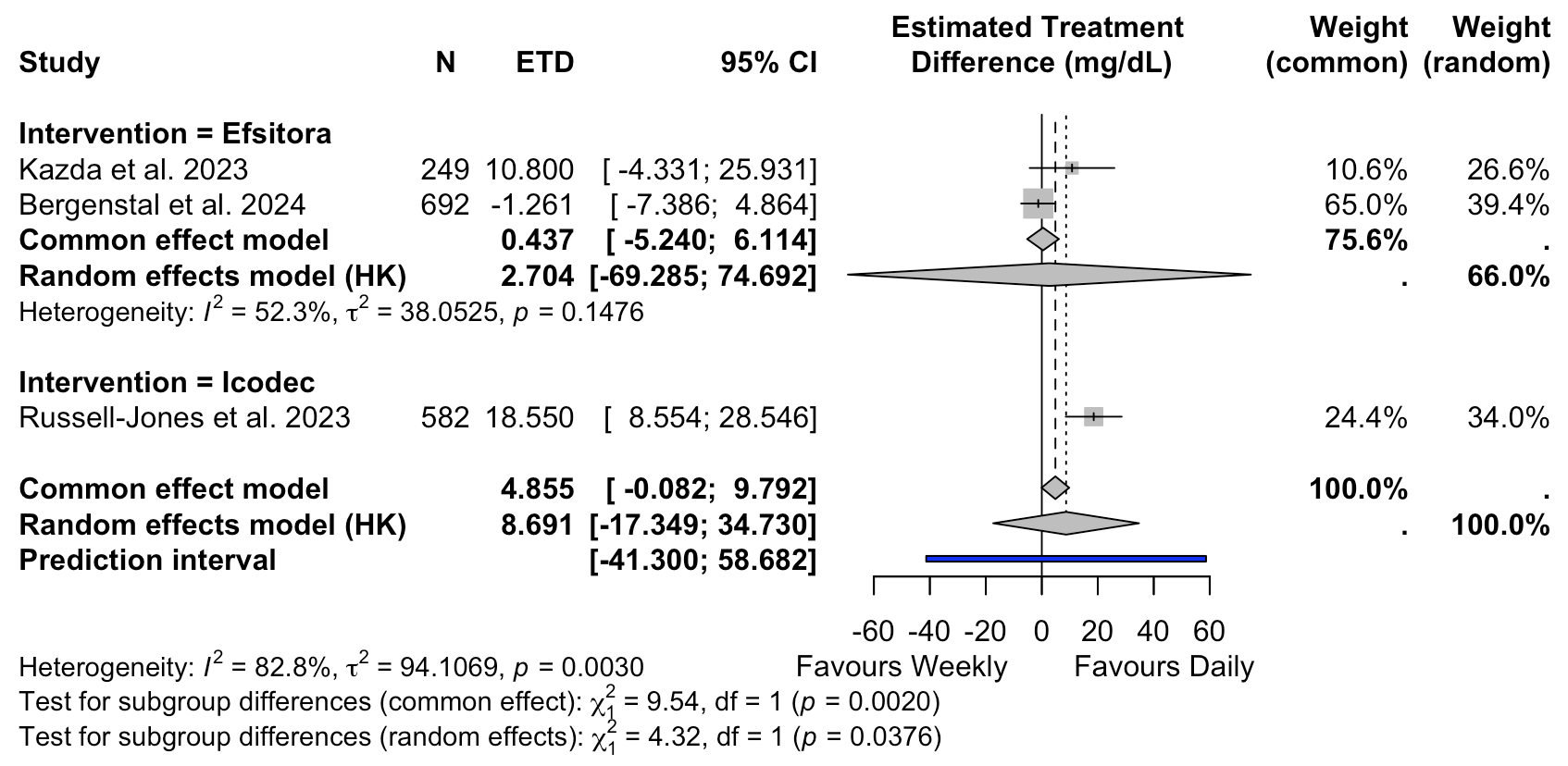


### 5.3 Body weight

###

###
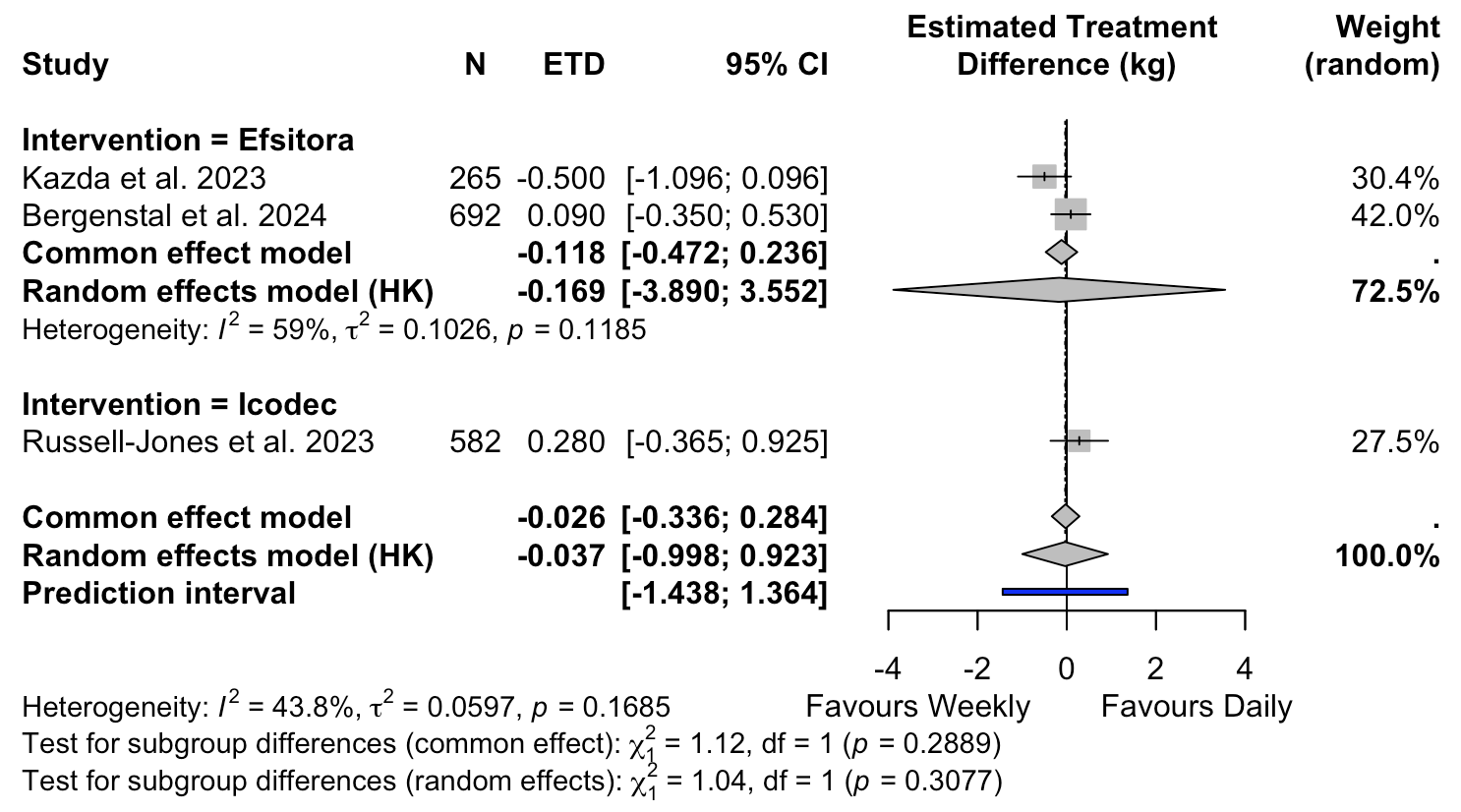


###

###

### 5.4 Time in range (70-180 mg/dL)

###

###
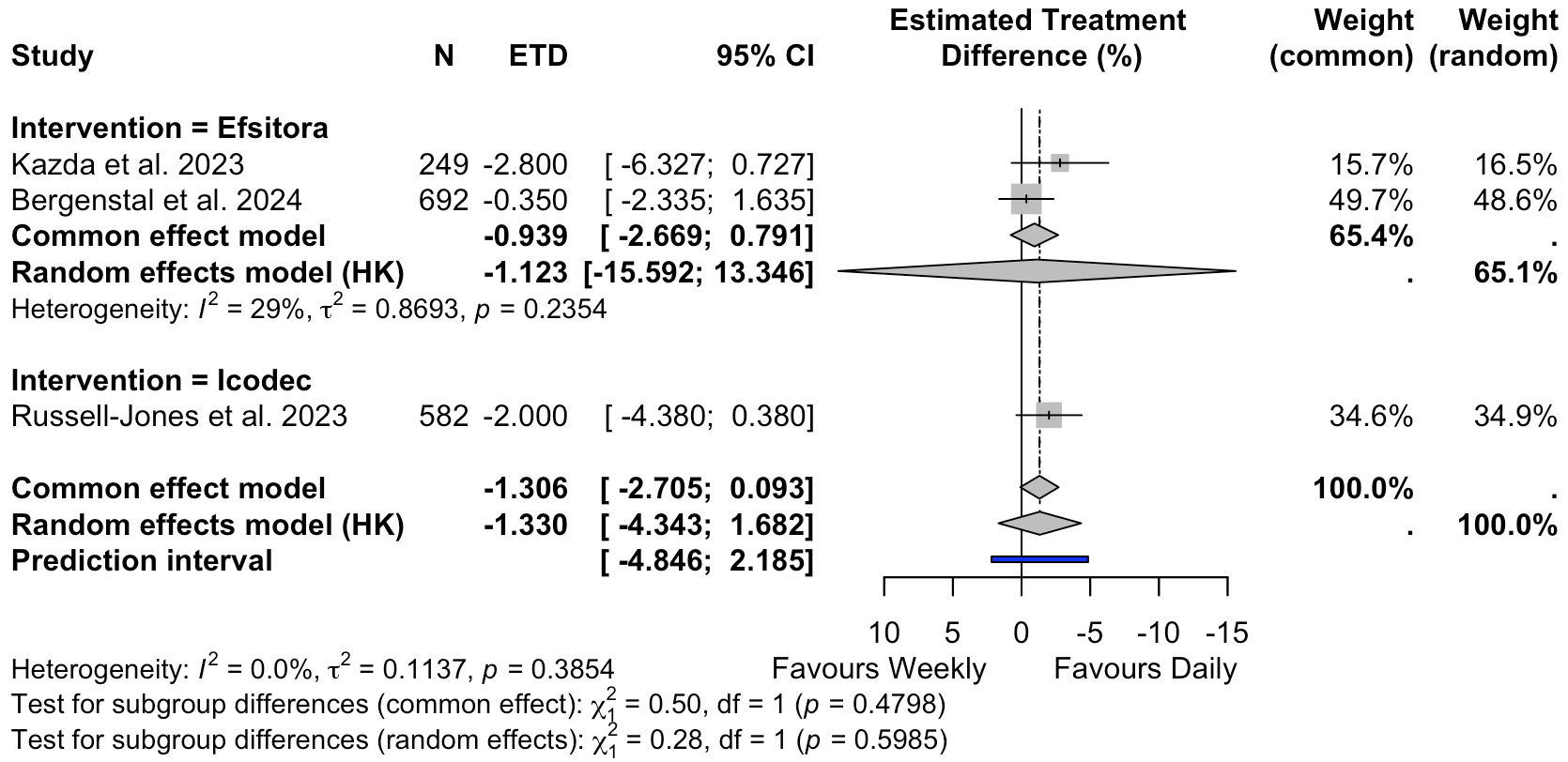


###

###

### 5.5 Time above range (>180 mg/dL)


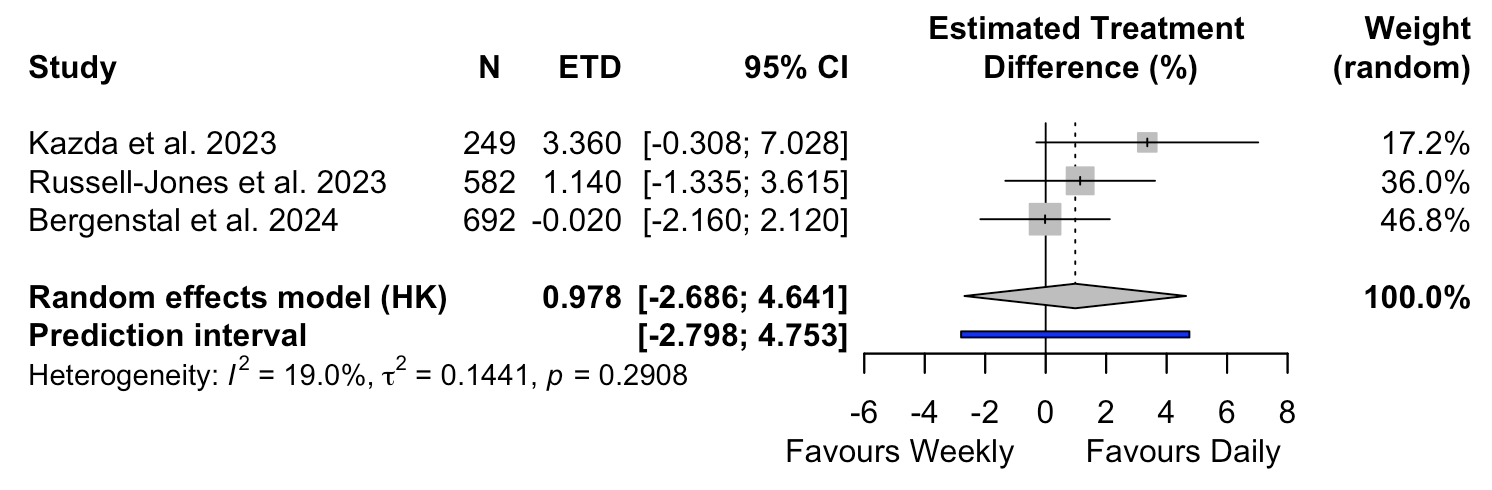


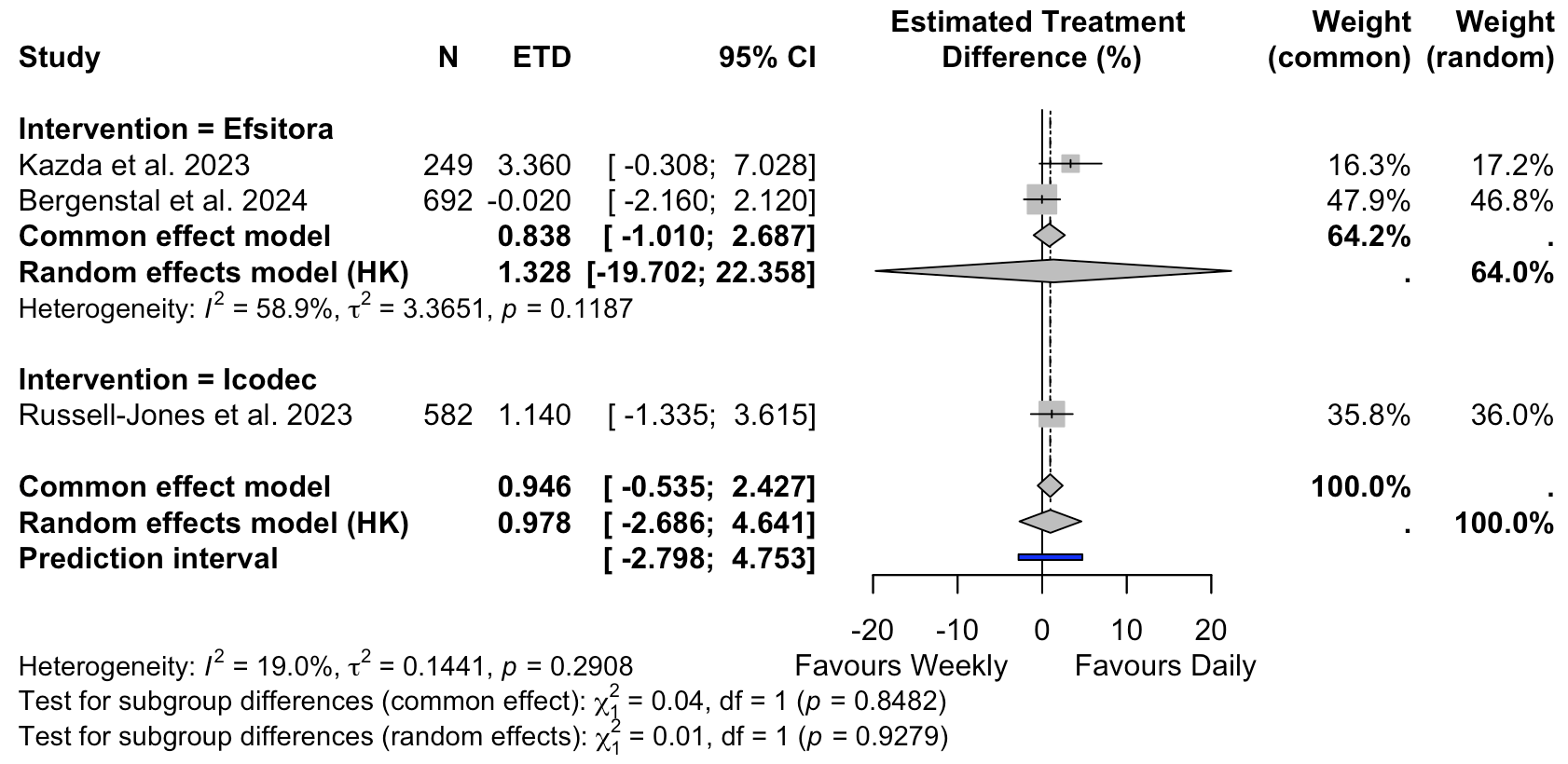


###

###

###

### 5.6 Time above range level 1 (181-250 mg/dL)

###

###
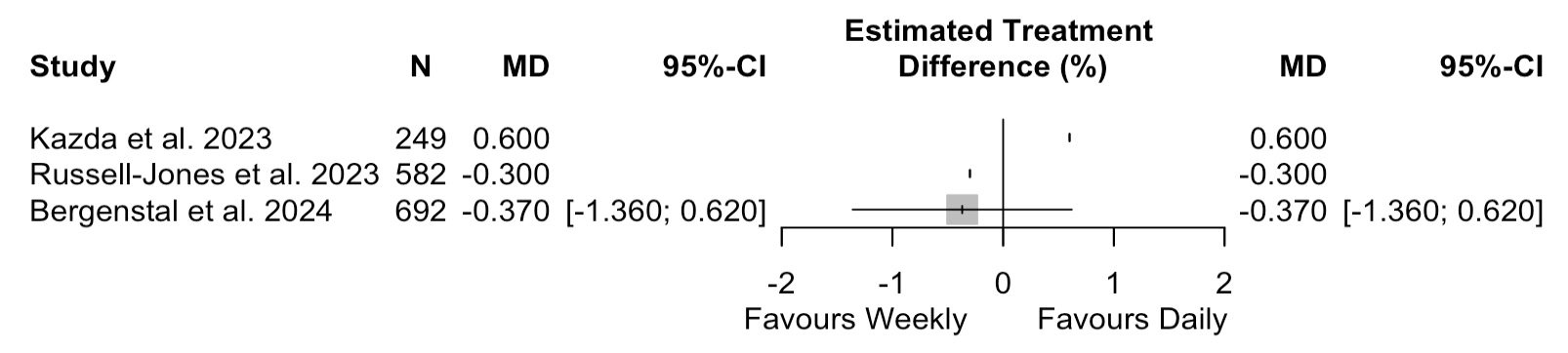


###

### 5.7 Time above range level 2 (>250 mg/dL)

###

###


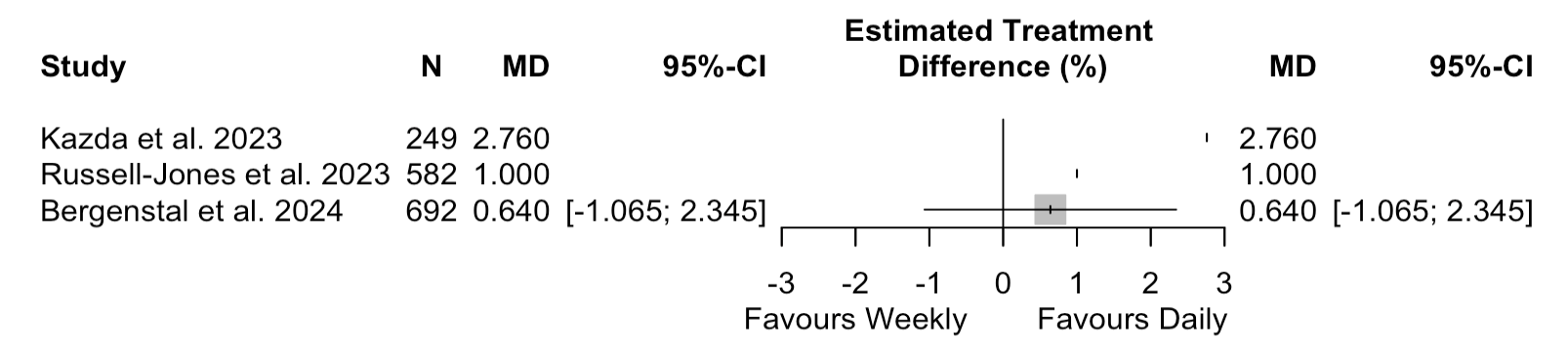


### 5.8 Time below range level 1 (54-69 mg/dL)


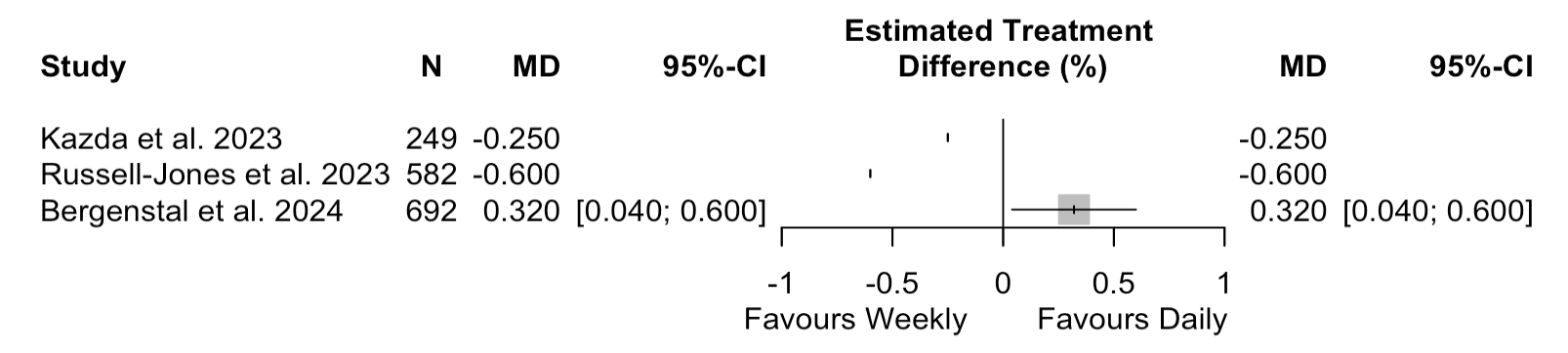


### 5.9 Time below range level 2 (<54 mg/dL)


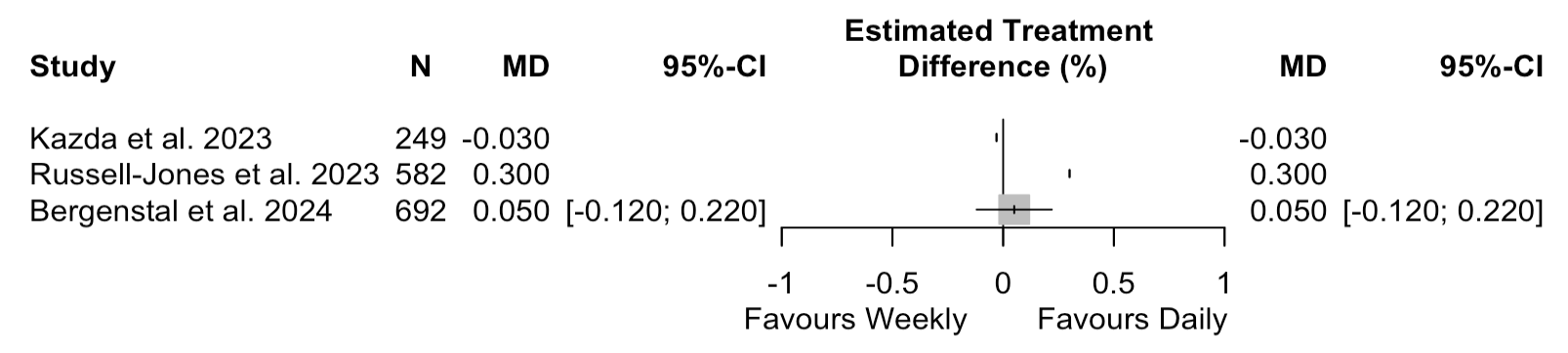


##

##

###

###

### 5.10 Level 1 hypoglycemic events


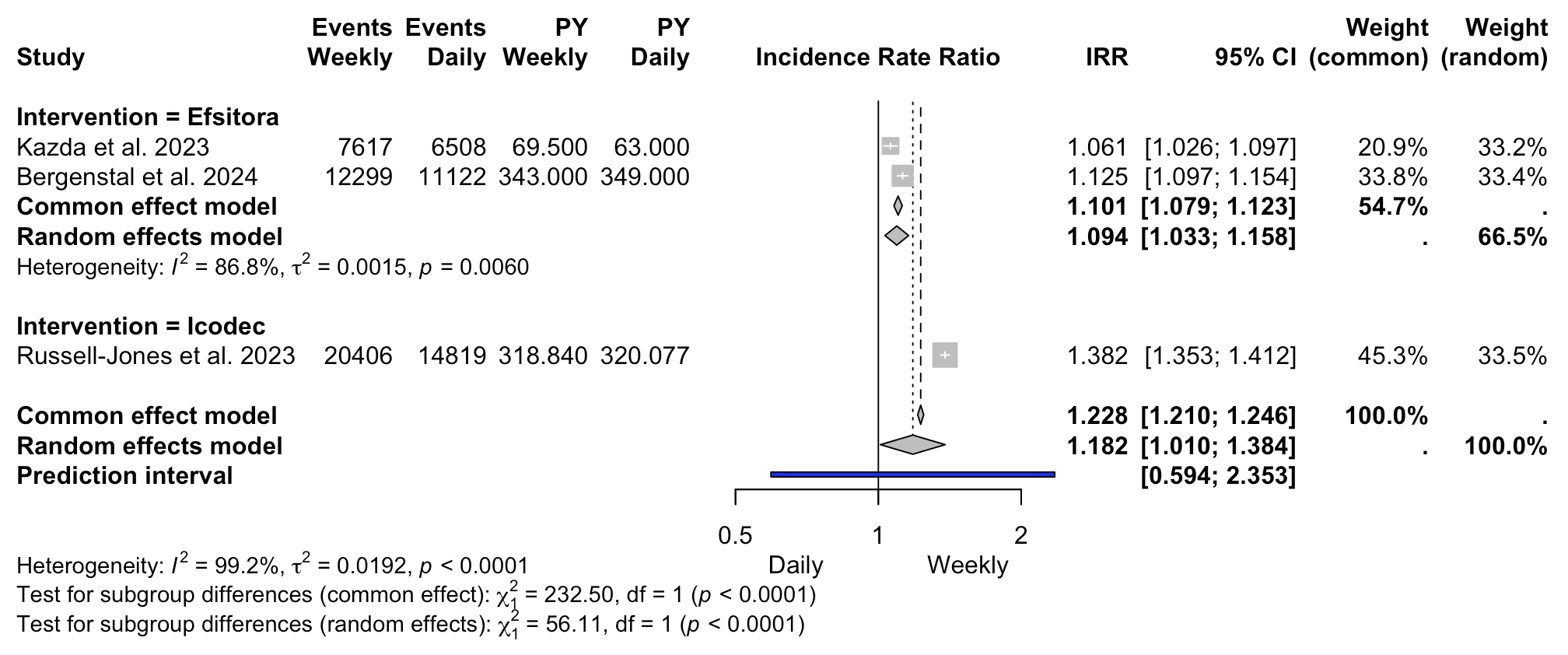


### 5.11 Level 2 hypoglycemic events

##
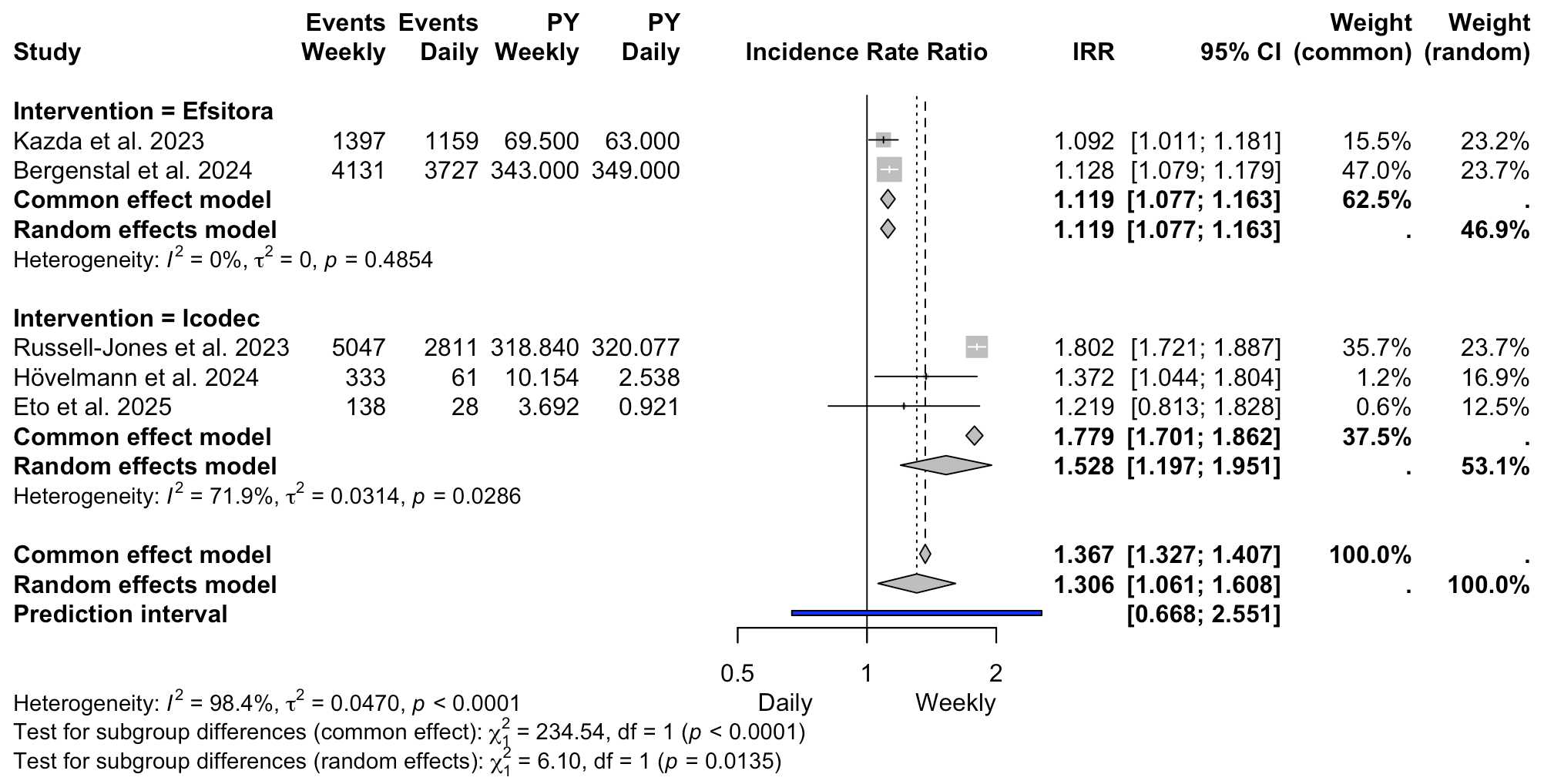


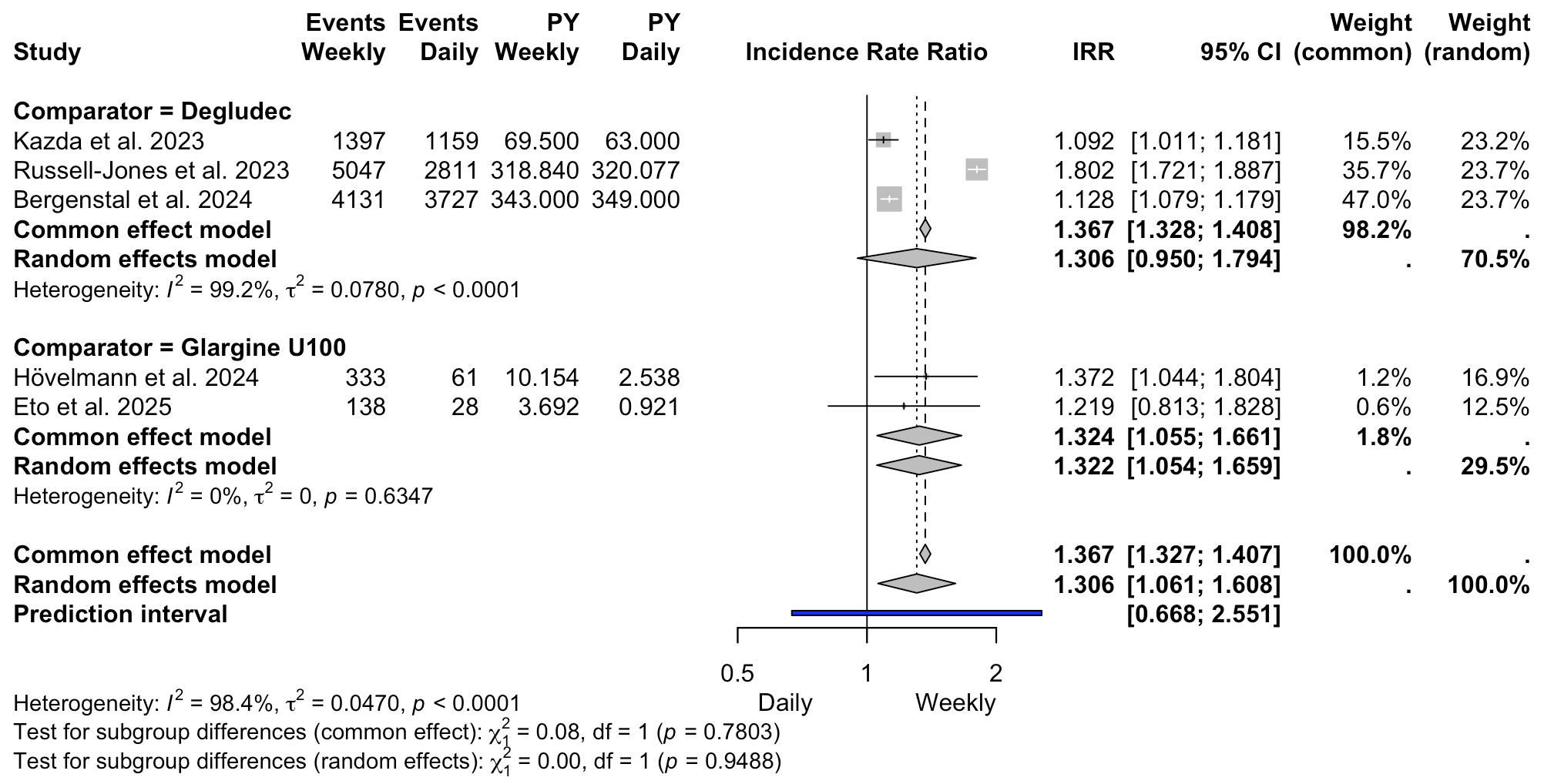


##

### 5.12 Level 3 hypoglycemic events

##

##
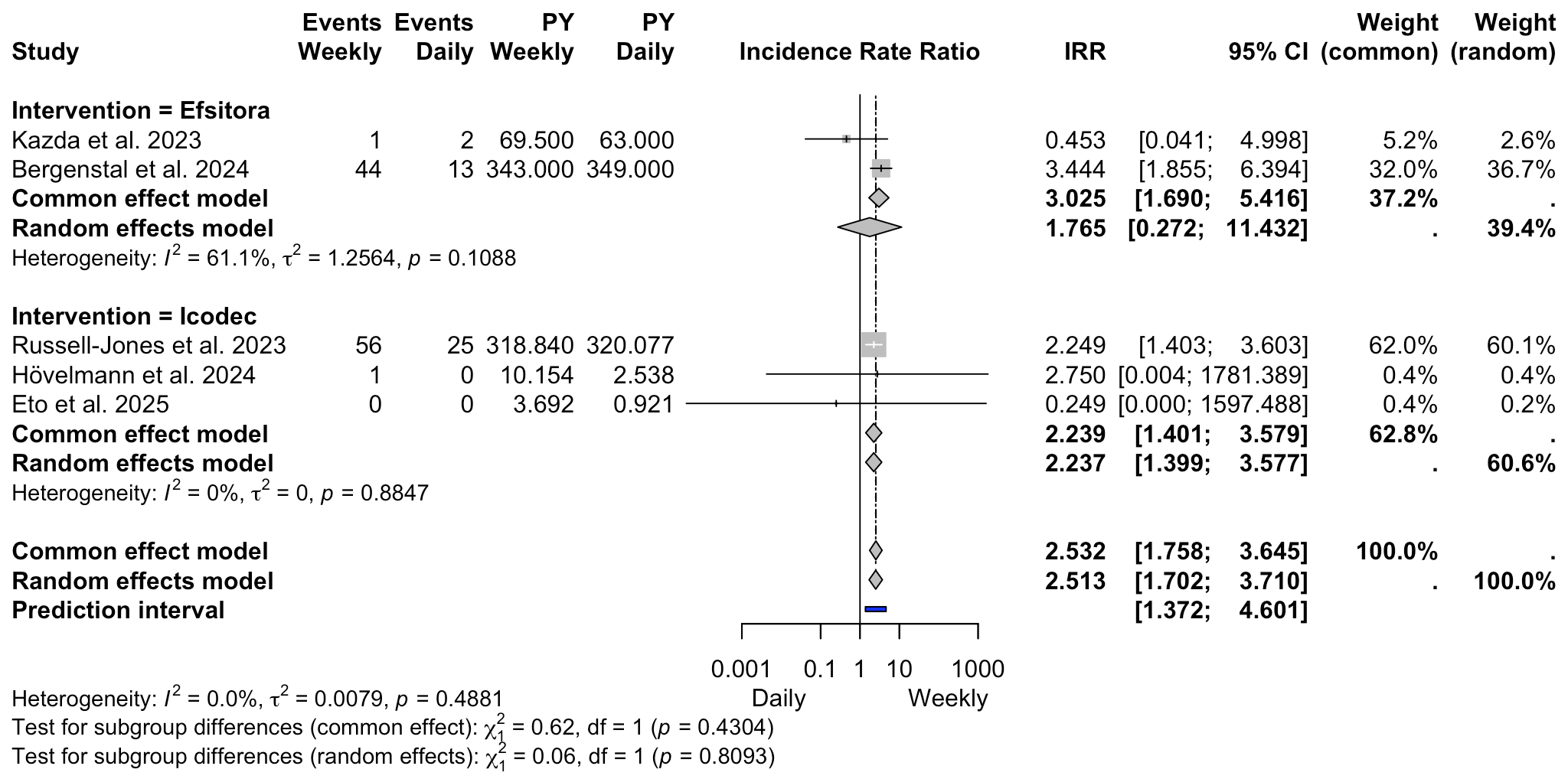


##

##
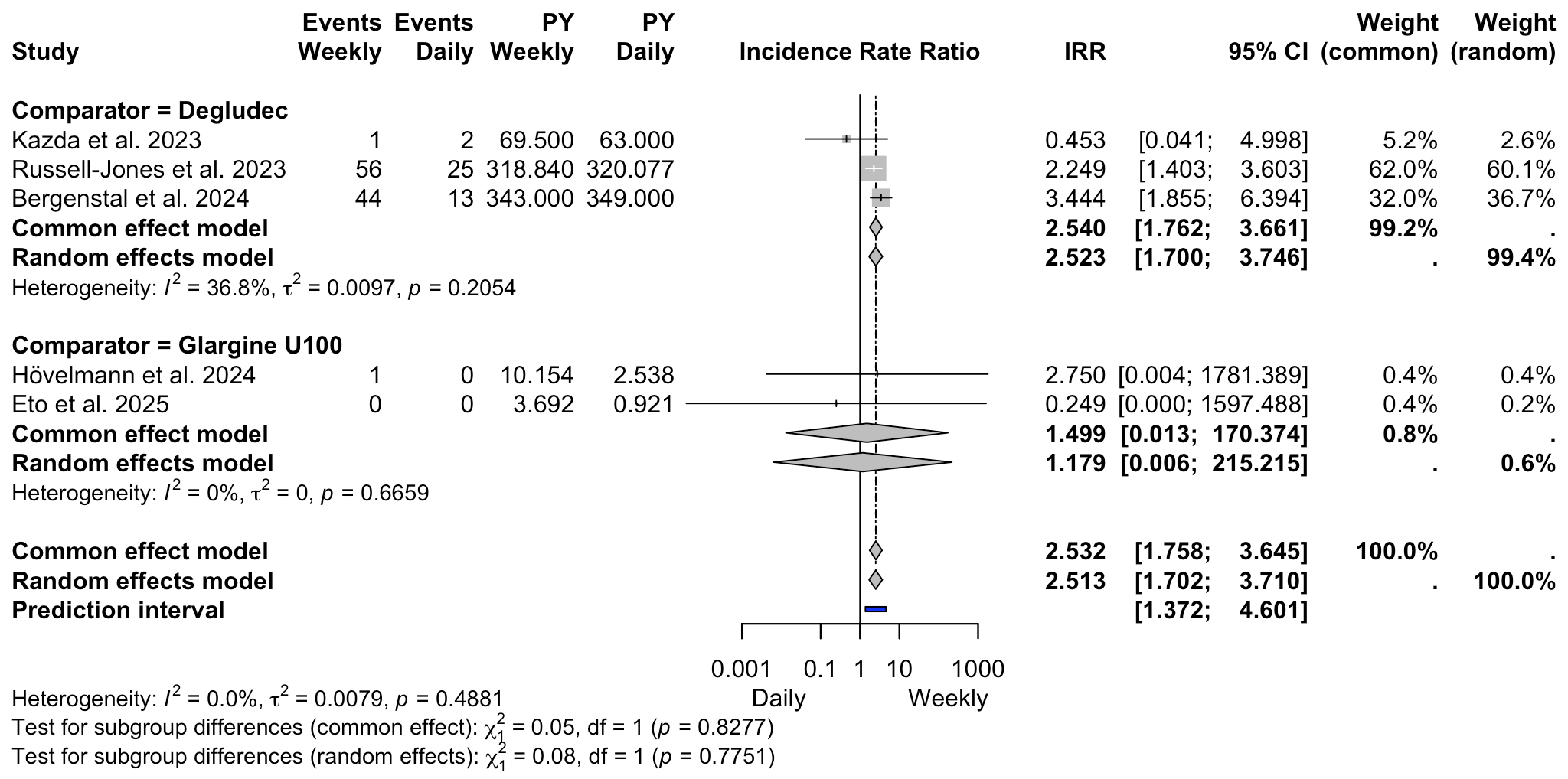


##
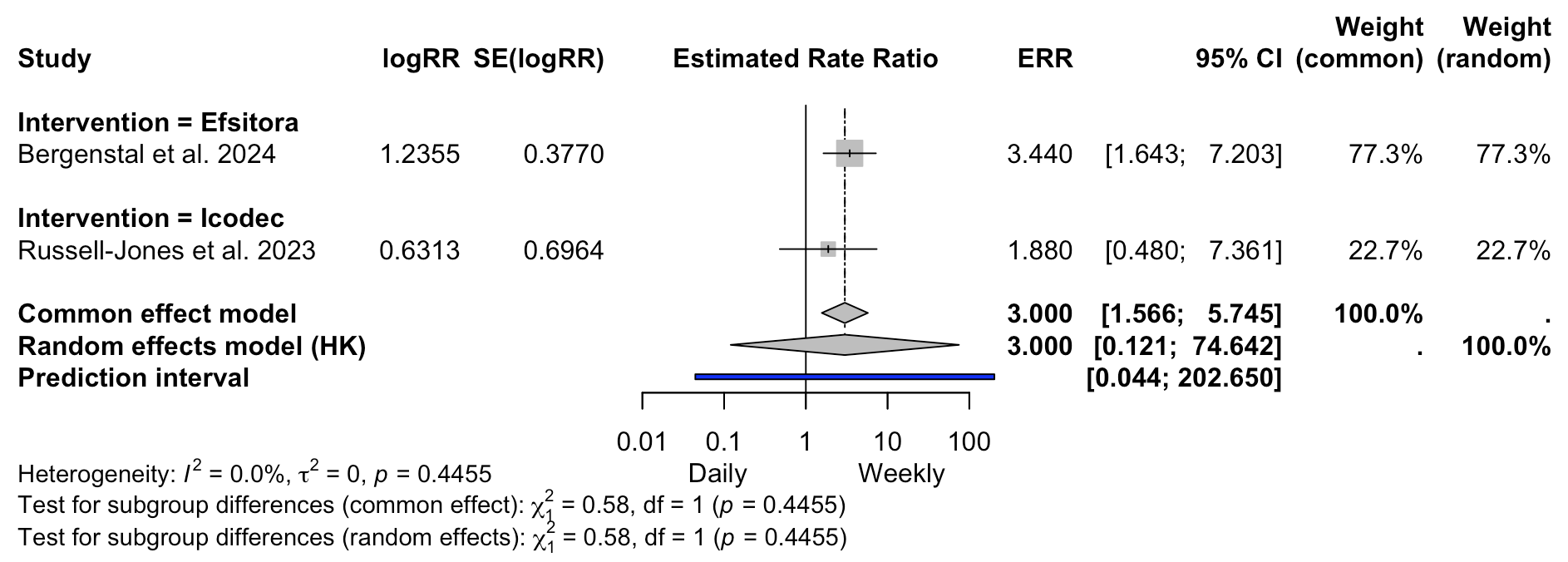


### 5.13 Total insulin dose

##


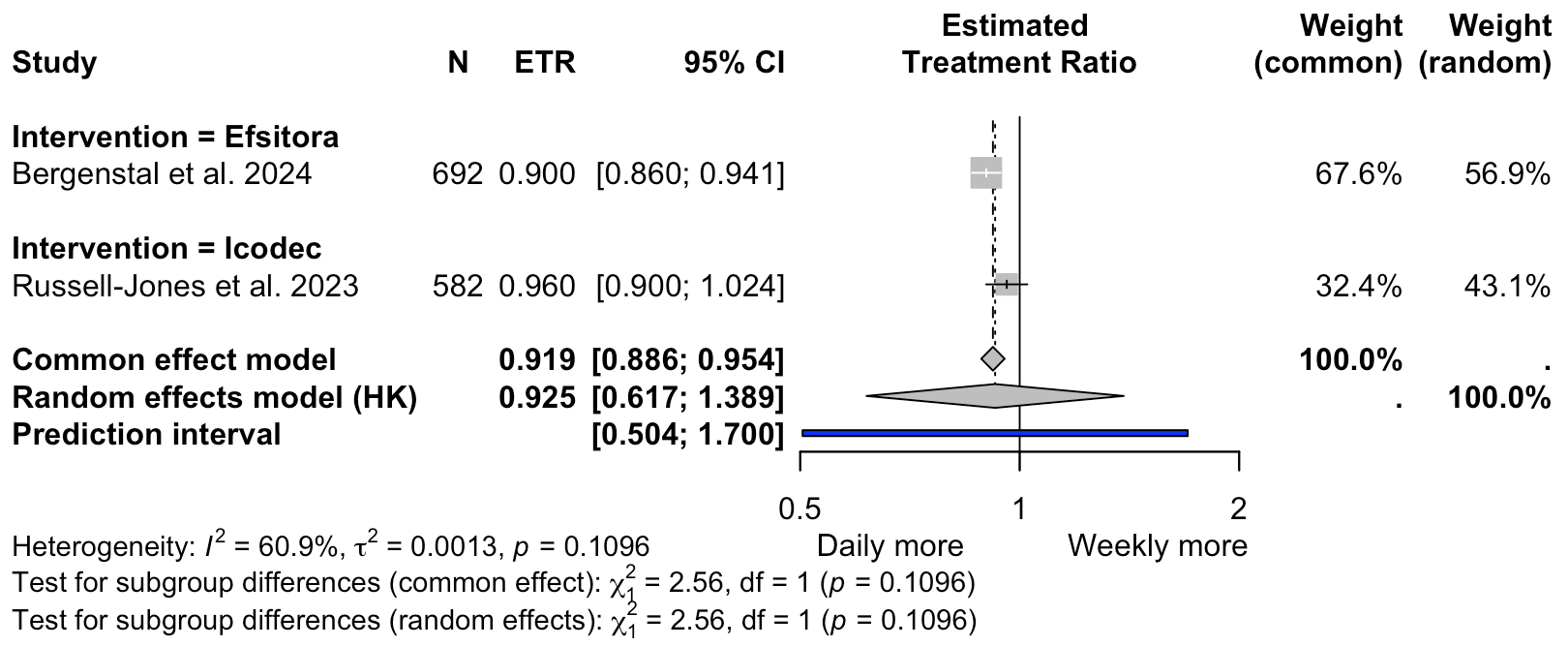


##

### 5.14 Basal insulin dose

##


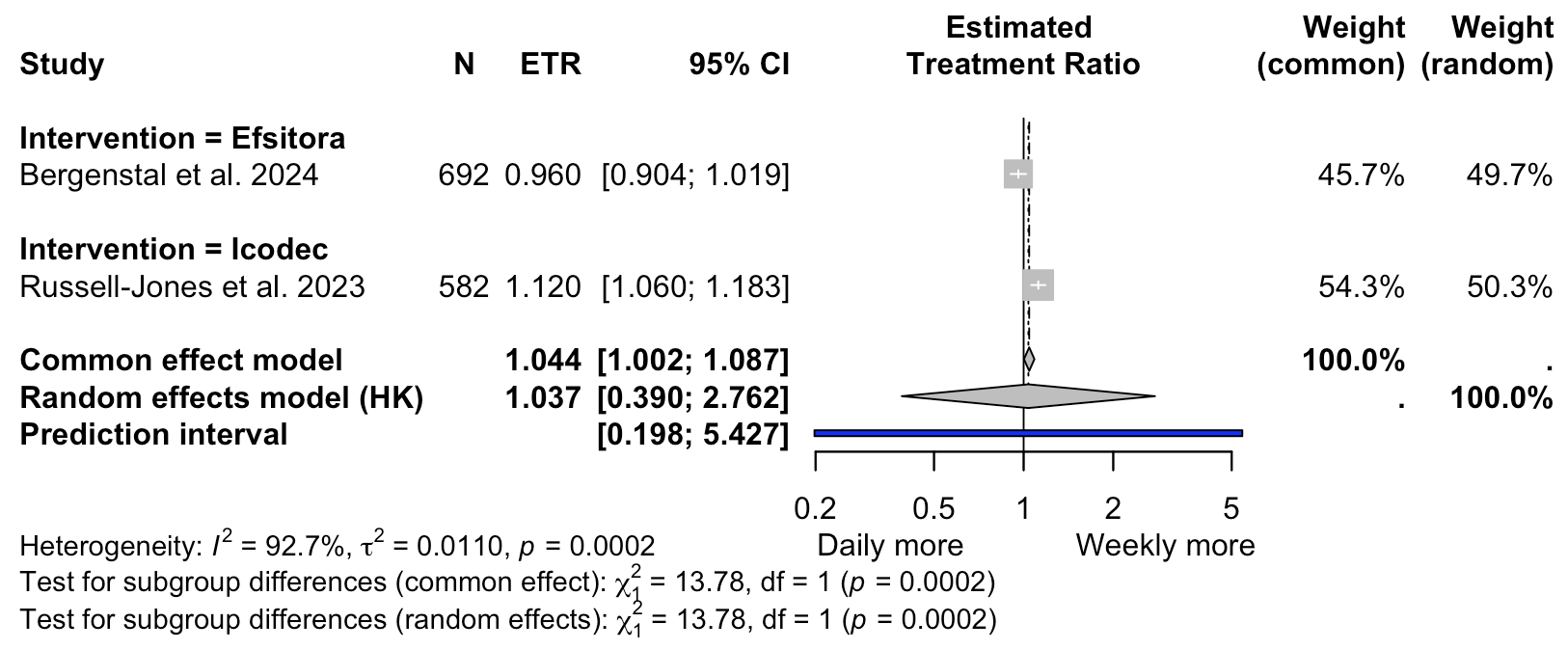


##

### 5.15 Bolus insulin dose

##


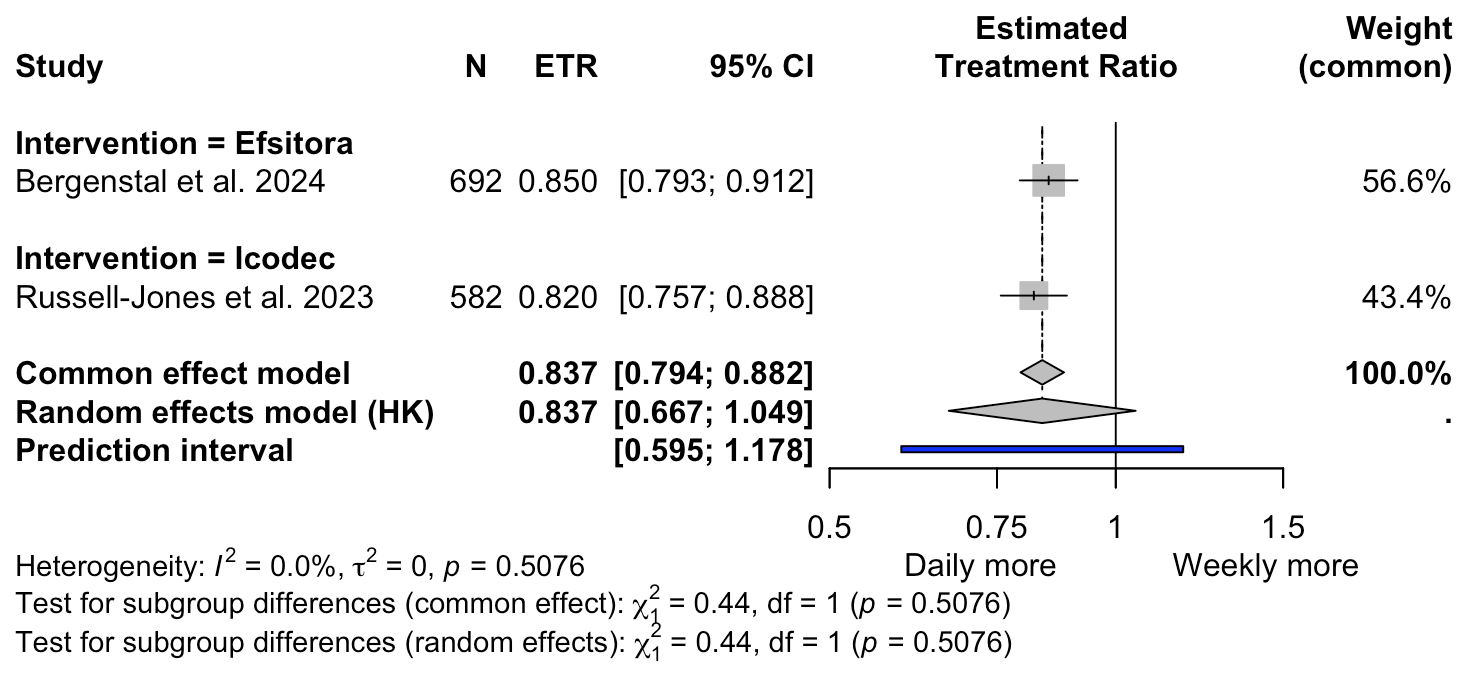


###

## Supplement 6. Summary of findings

| **Once-weekly basal insulin therapy in people with type 1 diabetes** | | | | | | |
| --- | --- | --- | --- | --- | --- | --- |
| **Patients or population:**people with type 1 diabetes treated with insulin injections  **Intervention:** weekly basal insulins  **Comparison:** any daily basal insulin  **Outcomes:** hemoglobin A1c (HbA1c), body weight and body mass index (BMI), mean daily basal insulin dose, total daily insulin dose, time spent in the 70-180 mg/dL glucose range (TIR), time spent below range (TBR), time spent above range (TAR), measures of glucose variability, patients’ satisfaction and quality of life, occurrence of confirmed and severe hypoglycemia, occurrence of diabetic ketoacidosis (DKA), cardiovascular outcomes  **Setting:** outpatients enrolled in randomized controlled trial | | | | | | |
| **Outcome** | **No of studies/**  **patients** | **Mean Difference**  **[95% CI]** | **Anticipated absolute effect** | **Relative effect** | **Certainty of evidence (GRADE)** | **Comment** |
| **HbA1c (%)** | 3/1523 | 0.083% [-0.009 to 0.175] | - | **-** | **⨁⨁⨁⨁ High** | Weekly and daily basal insulin have a similar effect on HbA1c |
| **TIR (%)** | 3/1523 | -1.306% [-2.705 to 0.093] | - | **-** | **⨁⨁⨁◯**  **Moderate^a^**  due to risk of bias | Weekly and daily basal insulins probably have a similar effect on TIR |
| **Body weight (kg)** | 3/1539 | -0.037 [-0.998 to 0.923] | - | **-** | **⨁⨁⨁◯**  **Moderate^b^**  due to inconsistency | Weekly and daily basal insulins probably have a similar effect on body weight |
| **Hypoglycemia Level 2**  **(incident rate ratio)** | 5/1719 | **-** | - | 1.31 [1.06-1.61] | **⨁◯◯◯ Very Low^c^**  due to inconsistency | The difference in the risk of level 2 hypoglycemia between weekly and daily basal insulin is uncertain |
| **Hypoglycemia Level 3**  **(incident rate ratio)** | 5/1719 | **-** | 4.5% more  (2.2 more to 7.7 more) | 2.53 [1.76-3.64] | **⨁⨁⨁◯**  **Moderate^d^**  due to risk of bias | Weekly basal insulin probably induce a greater risk of level 3 hypoglycemia compared to daily basal insulin |
| **Change in basal bolus units** | 2/1274 | 0.837  [0.79-0.88] | **-** | **-** | **⨁⨁⨁⨁ High** | Weekly basal insulin is associated with a lower bolus dose compared to daily basal insulin |
| **^a^**TIR evaluated on a 24h period in one study.  **^b^**I**^2^ =** 43.8%, although tau**^2^** low and non significant; visual inconsistency of point estimates.  **^c^**I**^2^ =** 98.4% and low overlap of confidence interval (overall confidence interval approaching the null effect)  **^d^**Unclear risk of bias for this outcome in 3 out of 5 studies  Anticipated absolute effect has not been calculated due to lack of information on the prevalence of hypoglycemia level 2 in the studies by Hovelman et al. and Eto et al. | | | | | | |

## Supplement 7. Other severe adverse events

| **Study** | **Kazda C.M., 2023** [(1)](https://paperpile.com/c/BO6zg2/BU3t8) | **Russell-Jones D., 2023 (ONWARDS 6)** [(2)](https://paperpile.com/c/BO6zg2/im1iT) | **Hövelmann U., 2024** [(3)](https://paperpile.com/c/BO6zg2/vM4bi) | **Bergenstal R.M., 2024 (QWINT-5)** [(4)](https://paperpile.com/c/BO6zg2/Nzqyn) | **Eto T., 2024** [(5)](https://paperpile.com/c/BO6zg2/he2xf) |
| --- | --- | --- | --- | --- | --- |
| **DKA, n (%)** | - | 1 (<1%) icodec *,  0 degludec | 0 | 0 efsitora,  2 (0.6%) degludec | 0 |
| **All cause death, n (%)** | 0 | - | 0 | 0 efsitora,  1 (0.3%) degludec * | 0 |
| **MACE, n (%)** | - | - | 0 | 0 efsitora,  2 (0.6%) degludec † | 0 |
| **AMI, n (%)** | - | - | 0 | 0 efsitora,  1 (0.3%) degludec | 0 |
| **Stroke, n (%)** | - | - | 0 | - | 0 |
| **Gangrene, n (%)** | - | - | 0 | - | 0 |
| **Lower limb**  **amputation, n (%)** | - | - | 0 | - | 0 |
| **Diabetic retinopahy, E (R)** | - | 28 (0.09) icodec,  29 (0.09) degludec | 0 | - | 0 |
| **DKD, n (%)** | - | - | 0 | - | 0 |
| **ESRD, n (%)** | - | - | 0 | - | 0 |

MACE: major adverse cardiovascular event; AMI: acute myocardial infarction; DKD: diabetic kidney disease; ESRD: end-stage renal disease; E: number of adverse events; R: rate (number of adverse events divided by person-year of exposure [one person-year of exposure=365.25 days]).

- not reported; * not treatment related; † coronary artery disease.

##

##

## References

1. [Kazda CM, Bue-Valleskey JM, Chien J, Zhang Q, Chigutsa E, Landschulz W, et al. Novel once-weekly basal Insulin Fc achieved similar glycemic control with a safety profile comparable to insulin degludec in patients with type 1 diabetes. Diabetes Care. 2023 May 1;46(5):1052–9.](http://paperpile.com/b/BO6zg2/BU3t8)

2. [Russell-Jones D, Babazono T, Cailleteau R, Engberg S, Irace C, Kjaersgaard MIS, et al. Once-weekly insulin icodec versus once-daily insulin degludec as part of a basal-bolus regimen in individuals with type 1 diabetes (ONWARDS 6): a phase 3a, randomised, open-label, treat-to-target trial. Lancet. 2023 Nov 4;402(10413):1636–47.](http://paperpile.com/b/BO6zg2/im1iT)

3. [Hövelmann U, Engberg S, Heise T, Kristensen NR, Nørgreen L, Zijlstra E, et al. Pharmacokinetic and pharmacodynamic properties of once-weekly insulin icodec in individuals with type 1 diabetes. Diabetes Obes Metab. 2024 May;26(5):1941–9.](http://paperpile.com/b/BO6zg2/vM4bi)

4. [Bergenstal RM, Weinstock RS, Mathieu C, Onishi Y, Vijayanagaram V, Katz ML, et al. Once-weekly insulin efsitora alfa versus once-daily insulin degludec in adults with type 1 diabetes (QWINT-5): a phase 3 randomised non-inferiority trial. Lancet. 2024 Sep 21;404(10458):1132–42.](http://paperpile.com/b/BO6zg2/Nzqyn)

5. [Eto T, Haranaka M, Kristensen NR, Navarria A, Nishida T, Ribel-Madsen R, et al. Pharmacological characteristics of once-weekly insulin icodec in Japanese individuals with type 1 diabetes. J Diabetes Investig. 2025 Mar;16(3):434–41.](http://paperpile.com/b/BO6zg2/he2xf)
